# Genetically and environmentally predicted obesity in relation to cardiovascular disease: a nationwide cohort study

**DOI:** 10.1016/j.eclinm.2023.101943

**Published:** 2023-04-06

**Authors:** Elsa Ojalehto, Yiqiang Zhan, Juulia Jylhävä, Chandra A. Reynolds, Anna K. Dahl Aslan, Ida K. Karlsson

**Affiliations:** a Department of Medical Epidemiology and Biostatistics, Karolinska Institutet, Stockholm, Sweden; b School of Public Health (Shenzhen), Sun Yat-Sen University, China; c Faculty of Social Sciences, Unit of Health Sciences and Gerontology Research Center, University of Tampere, Tampere, Finland; d Department of Psychology, University of California, Riverside, USA; e School of Health Sciences, University of Skövde, Skövde, Sweden

**Keywords:** Obesity, Cardiovascular disease, Polygenic score, BMI, Twins

## Abstract

**Background:**

Evidence indicates that the adverse health effects of obesity differ between genetically and environmentally influenced obesity. We examined differences in the association between obesity and cardiovascular disease (CVD) between individuals with a genetically predicted low, medium, or high body mass index (BMI).

**Methods:**

We used cohort data from Swedish twins born before 1959 who had BMI measured between the ages of 40–64 years (midlife) or at the age of 65 years or later (late-life), or both, and prospective CVD information from nationwide register linkage through 2016. A polygenic score for BMI (PGS_BMI_) was used to define genetically predicted BMI. Individuals missing BMI or covariate data, or diagnosed with CVD at first BMI measure, were excluded, leaving an analysis sample of 17,988 individuals. We applied Cox proportional hazard models to examine the association between BMI category and incident CVD, stratified by the PGS_BMI_. Co-twin control models were applied to adjust for genetic influences not captured by the PGS_BMI_.

**Findings:**

Between 1984 and 2010, the 17,988 participants were enrolled in sub-studies of the Swedish Twin Registry. Midlife obesity was associated with a higher risk of CVD across all PGS_BMI_ categories, but the association was stronger with genetically predicted lower BMI (hazard ratio from 1.55 to 2.08 for those with high and low PGS_BMI_, respectively). Within monozygotic twin pairs, the association did not differ by genetically predicted BMI, indicating genetic confounding not captured by the PGS_BMI_. Results were similar when obesity was measured in late-life, but suffered from low power.

**Interpretation:**

Obesity was associated with CVD regardless of PGS_BMI_ category, but obesity influenced by genetic predisposition (genetically predicted high BMI) was less harmful than obesity influenced by environmental factors (obesity despite genetically predicted low BMI). However, additional genetic factors, not captured by the PGS_BMI_, still influence the associations.

**Funding:**

The Strategic Research Program in Epidemiology at Karolinska Institutet; Loo and Hans Osterman’s Foundation; Foundation for Geriatric Diseases at Karolinska Institutet; the Swedish Research Council for Health, Working Life and Welfare; the 10.13039/501100004359Swedish Research Council; and the 10.13039/100000002National Institutes of Health.

**Research in context**

**Evidence before this study**

We searched PubMed for title and abstract keywords “((obesity) OR (adiposity) OR (BMI)) AND ((cardiovascular) OR (cardiac) OR (heart) OR (vascular)) AND ((polygenic) OR (genetic risk score) OR (genetic score))” through November 2022. The association between obesity and cardiovascular disease (CVD) is undisputable, and both phenotypes strongly influenced by genetic factors. However, some recent studies highlight that genetically predicted obesity may be less harmful than obesity influenced predominantly by non-genetic factors, such as environmental and lifestyle factors.

**Added value of this study**

By considering phenotypic obesity together with a polygenic score for body mass index (BMI; PGS_BMI_), we examined differences in CVD risk between a genetically predicted obesity versus obesity driven mainly by non-genetic factors. For these purposes, we used a cohort of almost 18,000 Swedish twins followed on average 18 years. There were indeed differences, with the risk increase for those with obesity influenced by lifestyle or other environmental factors (obesity despite a low PGS_BMI_) twice that of those with a genetically predicted obesity (obesity with a high PGS_BMI_; hazard rates 2.08 versus 1.55), compared to those with a healthy weight in the same PGS_BMI_ category. Utilizing the twin design of the data, we tested the associations within monozygotic twin pairs, who by default have the same genetically predicted BMI. Here, the association between obesity and CVD was substantially attenuated, and there were no differences in the association between those with genetically predicted low versus high BMI. This indicates that the association is still influenced by genetic factors, beyond the PGS_BMI_.

**Implications of all the available evidence**

While a healthy lifestyle is always to strive for, findings from the current study and previous work indicate that obesity influenced by environmental factors may be more deleterious than obesity influenced by genetic factors. The topic is still understudied, but this heterogeneity in obesity has been seen for several important outcomes, and in data from Sweden as well as the US. This, together with the attenuated association within twin pairs, indicates that the negative health effects of obesity may be mediated by other factors, rather than driven by the obesity in itself.

## Introduction

Over the last decades, the prevalence of overweight (body mass index (BMI) 25–29.9) and obesity (BMI ≥ 30) has increased worldwide to an extent that nearly a third of the population is classified as overweight or obese.^1^^,^^2^ This increase is alarming as it is well-established that a high BMI in midlife affects nearly all physiological functions and increases the risk of developing multiple disease conditions, including cardiovascular disease (CVD).^3^ In fact, two reviews and meta-analyses of Mendelian randomization studies have found that obesity is causally related to cardiovascular outcomes.^4^^,^^5^

Obesity and CVD are both complex phenotypes, influenced by genetic and environmental factors. Twin studies have estimated the heritability of BMI to 45–85%,^6^ and the most recent genome-wide association study (GWAS) identified 941 genetic variants underlying BMI,^7^ thus providing evidence that obesity and obesity-related phenotypes are eminently heritable. While a high BMI is understood to have both environmental and genetic influences,^8^ these influences have seldom been studied interactively. By simultaneously considering phenotypic BMI and its genetic influences, one can distinguish if a high BMI is influenced by genetic factors versus predominantly by non-genetic factors, such as environmental lifestyle factors. In fact, recent evidence indicates that genetically predicted overweight and obesity may be associated with lower disease risk than overweight and obesity driven by environmental factors.^9^, ^10^, ^11^, ^12^ We first demonstrated this in relation to dementia risk in the Swedish Twin Registry (STR), where a higher BMI in midlife was associated with a higher risk of dementia only among those with a genetically predicted low BMI (i.e. a higher BMI influenced by environmental factors, rather than genetic predisposition).^9^ The same has since been shown for cognitive abilities,^10^ mortality,^11^ and cardiovascular outcomes,^12^ all in data from the Health and Retirement Study.

However, differences between genetically versus environmentally influenced overweight and obesity are still understudied, and mainly based on the Health and Retirement Study data.^10^, ^11^, ^12^ Moreover, while the negative health effects of overweight and obesity in midlife are well-established, the causes and consequences of overweight or obesity in late-life are more complicated, with evidence of an inverse association with e.g. mortality.^13^ Thus, we aim to study how genetically predicted BMI interacts with phenotypic overweight and obesity to influence the risk of CVD, and if the associations differ depending on if overweight and obesity were measured in midlife or late-life. By using data from the STR, we will also examine if the associations hold within monozygotic (identical) twin pairs with the same genetically predicted BMI, thus accounting for other shared genetic and other familial factors.

## Methods

### Study population

**Fig. 1:**
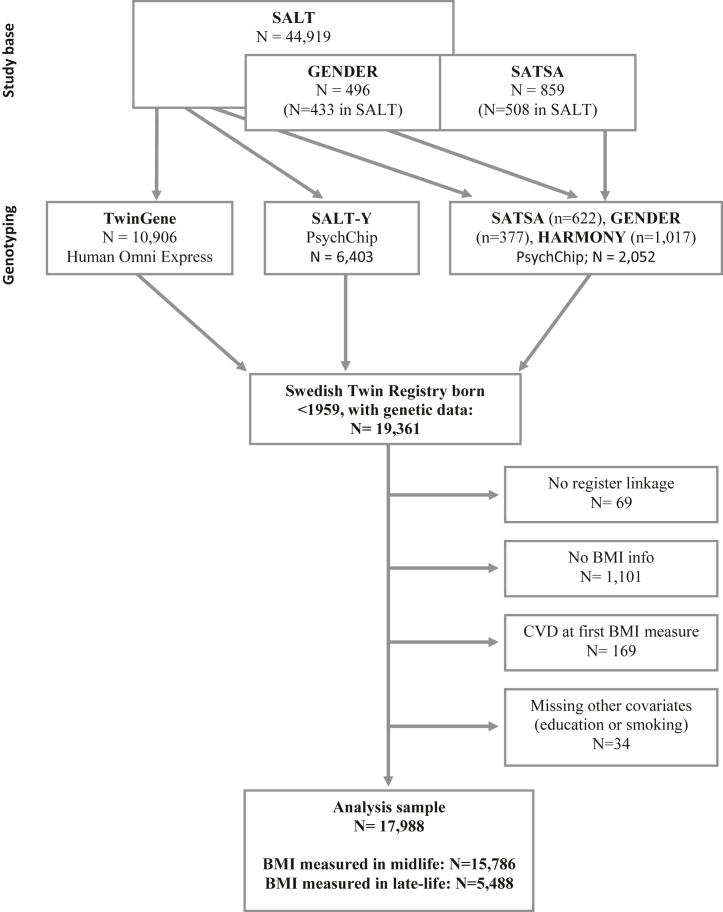
**Study profile.** Flow chart of the study population, originating from sub-studies of aging within the Swedish Twin Registry. ∗3286 individuals had measures taken in both age categories, and contributed to both sets of analyses.

The study population originates from the STR, a population-based register including virtually all twins born in Sweden.^14^ We used data from twins born 1958 or earlier, who participated in data collections that included genotyping (Fig. 1). Included sub-studies were the Swedish Adoption/Twin Study of Aging (SATSA),^15^ a study of 859 individuals from same-sex twin pairs followed-up in up to 10 waves of in-person testing conducted 1986–2014; Aging in Women and Men: A Longitudinal Study of Gender Differences in Health Behaviour and Health among Elderly (GENDER),^16^ a study of 496 individuals from opposite-sex twin pairs followed-up in three waves of in-person testing occurring 1995–2005; and the Screening Across the Lifespan Twin Study (SALT)^14^ which was aimed at all twins born 1958 or earlier, where 44,919 individuals participated in an extensive telephone interview. Genotyping data is available for a subset of SALT twins through participation in the sub-studies HARMONY^17^ (screening of cognitive functioning for SALT participants aged 65 and above, conducted 1998–2003), TwinGene^14^ (consisting of a questionnaire and a health checkup, conducted 2004–2008), and SALT-Y^14^ (consisting of a questionnaire and saliva collection, conducted 2009–2010).

In total, genotyping data is available for 19,361 individuals (Fig. 1). Sample characteristics for SATSA, GENDER, and SALT participants with and without genotyping data are shown in Supplementary Table S1. Those with genotyping data were on average born later (1942 versus 1939), younger at baseline (57 versus 61 years), older at death (80 versus 79 years), and had a lower proportion of individuals with low education (33% versus 67%) and smokers (42% versus 58%), compared to those without genotyping data. The latter difference in proportions likely reflect the later birth years of those with genotyping data.

#### Ethics

All participants provided informed consent and this study was approved by the Regional Ethical review Board in Stockholm (2015-1729-31-5) and the Swedish Ethical Review Authority (2022-06634-01). Additionally, this study followed the STROBE guidelines for cohort studies.^18^

### Cardiovascular disease information

The STR is linked to several nationwide registers,^14^ including the National Patient Registry (NPR) and the Cause of Death Registry (CDR) from where CVD information was retrieved in this study. The NPR reached nationwide coverage in 1987, and includes information about 99% of all overnight hospitalizations in Sweden.^19^ Since 2001 the NPR also includes outpatient specialist care. The CDR includes information about causes of death for all Swedish residents.^20^ Data were available throughout 2016. Primary and secondary diagnoses or causes of death are reported in ICD codes, and we used diagnostic codes for angina pectoris, myocardial infarction, atherosclerosis, claudication, ischemic heart disease, stroke, and the surgical procedures coronary artery by-pass graft and percutaneous transluminal coronary angioplasty, based on prior work in the STR.^21^ In addition to a broader CVD classification, we separated diagnoses into non-stroke CVD and stroke. Additional information about the registers along with ICD codes used to define any CVD, non-stroke CVD, and stroke is provided in the Supplementary section and Table S2.

### BMI measurement

Height and weight were measured in every data collection within SATSA, GENDER, HARMONY, and TwinGene, and self-reported as part of SALT. In addition, self-reported height and weight are available from questionnaires sent out to same-sex twin pairs in the 1960s and 70s.^14^ Twins born before 1926 were sent questionnaires in 1961, 1963, 1967, and 1970, which, in addition to height and weight at the time also included questions about their weight at ages 25 and 40. Twins born 1926–1958 were sent a questionnaire in 1973, with questions about height and weight at the time.

After carefully checking the data for outliers, both visually and quantitatively, BMI was calculated as kg/m^2^, and categorized into healthy weight (BMI 18.5–24.9) overweight (25–29.9), or obesity (BMI ≥ 30). Individuals with underweight (BMI < 18.5; due to low numbers) or BMI above 55 were excluded. For a detailed description of BMI data availability and cleaning, please see Karlsson et al. 2020.^9^ To examine the effect of midlife and late-life BMI separately, we selected the measure taken closest to age 50 (out of samples taken at age 40–64) and 75 (out of samples taken at age 65 or above) for each individual, respectively. Individuals with BMI measured taken in both age intervals could contribute to both age categories.

### Genetically predicted BMI

For a measure of genetically predicted BMI, we computed a polygenic score for BMI (PGS_BMI_) using the most recent genome-wide association study for BMI, which identified 941 genetic variants associated with BMI in data from ∼700,000 individuals.^7^ The significant variants together explained 6% of the trait variance, while a PGS using all genetic variants with p < 0.001 explained 14%.^7^ STR participants were part of the GWAS, and to avoid sample overlap new summary statistics were first computed, excluding the STR studies.

The STR participants were genotyped on Human OmniExpress (TwinGene, n = 10,906) or Illumina PsychArray (SATSA, GENDER, and HARMONY, which were genotyped together, n = 2052; and SALT-Y, n = 6403), and the data imputed against the Haplotype Reference Consortium reference panel.^22^ For the current study, we selected HapMap3 SNPs present on all three genotyping arrays with a minor allele frequency >1% and imputed with high quality (info score >0.8), resulting in 952,885 SNPs. To deal with linkage disequilibrium, effect size shrinkage with SBayesR^23^ was performed, and polygenic scores were then computed with Plink 2.0. Prior to analyses, the PGS_BMI_ was adjusted for genetic ancestry (by regressing out the first five principal components from the score) and standardized within genotyping data collection (TwinGene; SATSA, GENDER, and HARMONY; SALT-Y). Tertiles of the PGS_BMI_ were used to categorize individuals into having genetically predicted low, medium, or high BMI. Additional details regarding computation of the PGS_BMI_ are provided in the Supplementary section and Fig. S1.

### Statistical analyses

All analyses were conducted in STATA 17.0.^24^ Descriptive statistics of the total midlife and late-life sample as well as stratified by CVD status were selected to present the total number of individuals and percentage of the total for categorical variables, or mean and standard deviation for continuous variables. In each set of analyses, all individuals with relevant measures were included (Fig. 1). Midlife and late-life overweight and obesity were modelled separately in all models described below, with CVD diagnosis as the outcome. Cox proportional hazard models with attained age as the underlying time scale were selected for the main analyses, to appropriately account for age and time without requiring assumptions about the underlying baseline hazard. All models were conducted both in the total sample and separately in men and women. Measured BMI category (overweight or obesity, with healthy BMI as the reference category) and the PGS_BMI_ were modelled as exposures for the risk of incident CVD in Cox proportional hazard models, where individuals were followed from age at BMI measurement to CVD diagnosis, death, or end of follow-up, whichever occurred first. Individuals missing relevant variables were excluded.

We first modelled the independent effects of either BMI category or the PGS_BMI_ as predictors of CVD. Second, we conducted joint effect models, where both BMI category and the PGS_BMI_ were included, thus mutually adjusted. Third, we modelled interaction effects between BMI category and the PGS_BMI_. Last, to visualize differences in the effect of overweight and obesity between individuals with genetically predicted low, medium, or high BMI, we stratified the effect of BMI category by tertiles of the PGS_BMI_ (by including an interaction term between BMI category and tertiles of the PGS_BMI_ to obtain stratified effect sizes). Sex, education (coded as seven years or less versus more than seven years of education, corresponding to basic versus more than basic education at the time), and smoking (never versus ever smoker) were considered as time-invariant confounders in all models. To allow for different baseline hazard across studies, a categorical study variable was included in the strata statement (stratified Cox model). To account for relatedness among twins we applied robust standard errors. Examining correlations and variance inflation factors did not indicate issues of collinearity between BMI and the PGS_BMI_ or multicollinearity across variables (see Supplementary section). The proportional hazards assumption was examined visually by plotting survival curves by exposure and covariate levels, and statistically with the phtest command. The tests indicated non-proportional hazards in relation to BMI category, sex, and smoking, but without indications of substantially time-varying effects warranting closer examinations (Supplementary Fig. S2). We therefore used bootstrapping with n = 100 repetitions to obtain valid standard errors.^25^ Thus, hazard ratios represent weighted average across follow-up time.

To test if the association between BMI category and CVD is explained by genetic factors not captured by the PGS_BMI_, we conducted co-twin control analyses. In such analyses, twin individuals (within a pair) who are discordant for the outcome are compared in relation to their exposure status.^26^ As twins share both DNA (to a varying extent), in utero environment, and early life environment, the co-twin control design elegantly controls for such confounding. In a Cox model, this is done by using each twin pair as a strata in the stratified Cox model, thus examining the exposure-outcome association within each pair. The baseline hazard is thereby allowed to differ between twin pairs, while the resulting estimates are optimized to fit all strata, and represent the within-twin pair estimates, adjusted for factors shared between twins. We first repeated the models above in co-twin control models of dizygotic (fraternal) twin pairs, sharing on average 50% of their co-segregating genes and thus differing in their PGS_BMI_. Second, we conducted co-twin control analyses of the association between BMI category and CVD risk within monozygotic twin pairs, sharing identical DNA. As co-twin control models rely on differences within twin pairs, models including the PGS_BMI_ cannot be applied within monozygotic twin pairs (as they, by default, have identical PGS_BMI_). However, by stratifying the sample into monozygotic twin pairs with genetically predicted low, medium, or high BMI, we could study the within monozygotic pair association between BMI category and CVD risk, by tertiles of the PGS_BMI_ (i.e. comparable to the last model described for the main analysis). The co-twin control models included education, sex, and smoking as covariates.

#### Sensitivity analyses

We tested if the associations are affected by survival bias by conducting competing risk regression with CVD as the outcome and death as the competing risk, with sex, smoking, education, and study as covariates. We also separately modelled non-stroke CVD and stroke in survival analysis set up in the same manner as the main models, to test for subtype-specific effects. When modelling non-stroke CVD, individuals were censored at the age of a stroke diagnosis.

#### Post-hoc analyses

We finally tested the associations in the full analysis sample, regardless of age at measurement, using the first available measure after age 40. Survival analysis was set up in the same manner as the main models also for the post-hoc analysis.

### Role of the funding source

The funders had no role in study design, data collection, data analysis, interpretation, or writing of the manuscript.

## Results

### Population characteristics

The participants were enrolled in respective sub-studies between 1984 and 2010, resulting in 19,361 individuals with genetic data (Fig. 1). After removing individuals who lacked register linkage (n = 69), had no BMI information (n = 1101), were diagnosed with CVD already at first BMI measurement (n = 169), or were missing for covariate information (n = 34), 17,988 individuals remained for analyses. Out of those, 15,786 individuals had BMI measured in midlife and 5488 had BMI measured in late-life. 3286 had measures taken in both age categories, and contributed to both sets of analyses. A flowchart of the study population is provided in Fig. 1 and sample characteristics in Table 1.

**Table 1:** Descriptive statistics of the study population.

	Midlife			Late life		
	All	No CVD	CVD	All	No CVD	CVD
Female sex, N (%)	8493 (53.80)	7172 (56.64)	1321 (42.30)	2918 (53.17)	2132 (56.00)	786 (46.76)
Male sex, N (%)	15,786 (46.20)	12,663 (43.36)	3123 (57.70)	5488 (46.83)	3807 (44.00)	1681 (53.24)
Low education, N (%)	3248 (20.58)	2279 (18.00)	969 (31.03)	1763 (32.12)	1113 (29.24)	650 (38.67)
Smokers, N (%)	9604 (60.84)	7691 (60.74)	1913 (61.26)	2758 (50.26)	1886 (49.54)	872 (51.87)
Age at baseline, M (SD)	52.08 (5.49)	52.20 (5.25)	51.60 (6.36)	71.76 (5.14)	71.00 (4.99)	73.48 (5.04)
Age at last follow up, M (SD)	71.18 (8.67)	69.67 (7.79)	77.33 (9.33)	82.18 (6.23)	81.30 (5.99)	84.16 (6.31)
Age at death, M (SD)	79.76 (10.18)	77.48 (10.85)	81.84 (9.05)	84.17 (7.07)	83.29 (7.39)	85.13 (6.58)
BMI at baseline, M (SD)	24.90 (3.33)	24.80 (3.33)	25.31 (3.30)	25.94 (3.74)	25.85 (3.78)	26.15 (3.66)

Descriptive statistics for all individuals, individuals without CVD and individuals with CVD, and BMI measured in midlife or late-life. Statistics are presented as number (%) of individuals for categorical variables and mean level (SD) for continuous variables. *BMI* Body Mass Index, *M* mean, *N* number, *SD* standard deviation.

The midlife sample was followed during 278,705 (mean 17.7, range 1–62) person-years, during which 3123 individuals were diagnosed with CVD at mean age 70.0 years. A total of 2414 individuals were diagnosed with non-stroke CVD at mean age 70.1 years, and 1156 with stroke at mean age 72.6 years. The late-life sample was followed during 50,614 (mean 9.2, range 1–36) person-years, during which 1681 individuals were diagnosed with CVD at mean age 80.2 years. A total of 1246 individuals were diagnosed with non-stroke CVD at mean age 80.6 years, and 831 with stroke at mean age 80.8 years.

The midlife sample included 5144 complete twin pairs (3124 dizygotic and 2020 monozygotic pairs), out of whom 1212 (769 dizygotic and 443 monozygotic pairs) were discordant for CVD. The late-life sample included 1670 complete twin pairs (1235 dizygotic and 435 monozygotic pairs), out of whom 565 (436 dizygotic and 129 monozygotic pairs) were discordant for CVD.

One standard deviation (SD) higher PGS_BMI_ was associated with 1.12 (95% CI 1.07–1.18) units higher BMI in the midlife sample and 1.16 (95% CI 1.07–1.26) units higher BMI in the late-life sample, after adjusting for sex and age at BMI measure. Additional adjustment for smoking and education did not affect the estimates.

### Midlife BMI category and the PGS_BMI_ in relation to risk of CVD

#### Independent and joint effect models of BMI category and the PGS_BMI_

In the independent effect models, midlife overweight and obesity were associated with a 31% and 76% higher risk of CVD, respectively (Table 2). One SD higher PGS_BMI_ was associated with a 12% higher risk of CVD. In the joint effect models, all estimates were slightly attenuated but remained statistically significant (Table 2). The association between obesity and CVD was slightly stronger in women than in men, but the results were overall comparable across sexes.

**Table 2:** Risk of CVD in relation to midlife and late-life BMI category and the PGS_BMI_, in the total sample, by sex, and within dizygotic twin pairs.

Midlife	Total sample			Within dizygotic twin pairs
	All	Men	Women	
**Independent effect model**				
Overweight	**1.31 (1.22–1.42), p < 0.001**	**1.31 (1.19–1.45), p < 0.001**	**1.29 (1.15–1.45), p < 0.001**	**1.35 (1.10–1.66), p = 0.004**
Obesity	**1.76 (1.50–2.05), p < 0.001**	**1.65 (1.36–2.00), p < 0.001**	**1.87 (1.53–2.28), p < 0.001**	**1.64 (1.11–2.42), p = 0.013**
PGS_BMI_	**1.12 (1.08–1.16), p < 0.001**	**1.12 (1.07–1.17), p < 0.001**	**1.12 (1.06–1.18), p < 0.001**	**1.16 (1.02–1.32), p = 0.027**
**Joint effect model**				
Overweight	**1.27 (1.18–1.38), p < 0.001**	**1.27 (1.15–1.40), p < 0.001**	**1.26 (1.12–1.42), p < 0.001**	**1.31 (1.06–1.62), p = 0.012**
Obesity	**1.64 (1.40–1.93), p < 0.001**	**1.53 (1.25–1.87), p < 0.001**	**1.76 (1.42–2.19), p < 0.001**	**1.55 (1.04–2.30), p = 0.031**
PGS_BMI_	**1.07 (1.03–1.11), p < 0.001**	**1.07 (1.02–1.13), p = 0.006**	**1.06 (1.00–1.13), p = 0.046**	1.10 (0.97–1.26), p = 0.145
**Interaction model**				
Overweight	**1.26 (1.17–1.37), p < 0.001**	**1.26 (1.14–1.40), p < 0.001**	**1.25 (1.10–1.41), p < 0.001**	**1.31 (1.06–1.62), p = 0.012**
Obesity	**1.83 (1.53–2.18), p < 0.001**	**1.69 (1.36–2.11), p < 0.001**	**1.96 (1.56–2.47), p < 0.001**	**1.69 (1.05–2.74), p = 0.032**
PGS_BMI_	**1.08 (1.03–1.13), p = 0.002**	1.07 (0.99–1.17), p = 0.093	**1.08 (1.00–1.16), p = 0.048**	1.15 (0.98–1.35), p = 0.089
Overweight ∗ PGS_BMI_	1.02 (0.95–1.09), p = 0.640	1.02 (0.90–1.15), p = 0.742	1.01 (0.88–1.15), p = 0.921	0.93 (0.76–1.14), p = 0.490
Obesity ∗ PGS_BMI_	**0.86 (0.74–1.00), p = 0.046**	0.87 (0.71–1.06), p = 0.163	0.85 (0.70–1.04), p = 0.111	0.85 (0.58–1.23), p = 0.379


Late life	Total sample			Within dizygotic twin pairs
	All	Men	Women	
**Independent effect model**				
Overweight	**1.22 (1.09–1.36), p = 0.001**	**1.31 (1.13–1.52), p < 0.001**	1.10 (0.96–1.26), p = 0.171	1.30 (0.97–1.74), p = 0.081
Obesity	**1.40 (1.21–1.62), p < 0.001**	**1.44 (1.16–1.79), p = 0.001**	**1.34 (1.11–1.62), p = 0.002**	1.42 (0.92–2.21), p = 0.115
PGS_BMI_	**1.09 (1.04–1.14), p < 0.001**	**1.13 (1.06–1.20), p < 0.001**	1.05 (0.98–1.12), p = 0.187	1.11 (0.93–1.33), p = 0.256
**Joint effect models**				
Overweight	**1.19 (1.06–1.34), p = 0.003**	**1.27 (1.10–1.47), p = 0.001**	1.09 (0.95–1.26), p = 0.224	1.28 (0.95–1.72), p = 0.107
Obesity	**1.33 (1.15–1.55), p < 0.001**	**1.33 (1.06–1.67), p = 0.014**	**1.32 (1.08–1.62), p = 0.008**	1.37 (0.87–2.16), p = 0.174
PGS_BMI_	**1.06 (1.01–1.11), p = 0.021**	**1.09 (1.03–1.17), p = 0.006**	1.02 (0.95–1.10), p = 0.619	1.07 (0.88–1.29), p = 0.503
**Interaction model**				
Overweight	**1.19 (1.06–1.33), p = 0.004**	**1.26 (1.09–1.46), p = 0.002**	1.09 (0.94–1.25), p = 0.261	1.23 (0.91–1.66), p = 0.184
Obesity	**1.40 (1.19–1.64), p < 0.001**	**1.41 (1.07–1.85), p = 0.014**	**1.37 (1.10–1.70), p = 0.004**	**1.74 (1.04–2.93), p = 0.036**
PGS_BMI_	**1.08 (1.00–1.17), p = 0.046**	1.11 (1.00–1.24), p = 0.053	1.04 (0.93–1.17), p = 0.460	1.20 (0.93–1.54), p = 0.161
Overweight ∗ PGS_BMI_	0.98 (0.88–1.10), p = 0.787	0.99 (0.85–1.15), p = 0.862	0.98 (0.85–1.13), p = 0.812	0.91 (0.66–1.24), p = 0.538
Obesity ∗ PGS_BMI_	0.89 (0.77–1.04), p = 0.134	0.88 (0.67–1.16), p = 0.354	0.91 (0.77–1.07), p = 0.263	**0.56 (0.34–0.92), p = 0.021**

Hazard rate ratios (95% confidence intervals) of CVD in relation to midlife or late-life BMI category and the PGS_BMI_, for the total sample, separately for men and women, and from co-twin control analyses of dizygotic twin pairs. All models are adjusted for study, sex, smoking and education, and age used as the underlying time scale. Independent effect models contain either BMI category *or* PGS_BMI_ as predictors of CVD. Joint effect models contain BMI category and PGS_BMI_ together as predictors of CVD. Interaction models contain main effects of BMI category and the PGS_BMI_, and an interaction term between BMI category and the PGS_BMI_. Statistically significant estimates (at the α < 0.05 level) are presented in bold. *BMI* body mass index, *CVD* cardiovascular disease, *PGS* polygenic score.

#### Interactive effects between BMI category and the PGS_BMI_

**Fig. 2:**
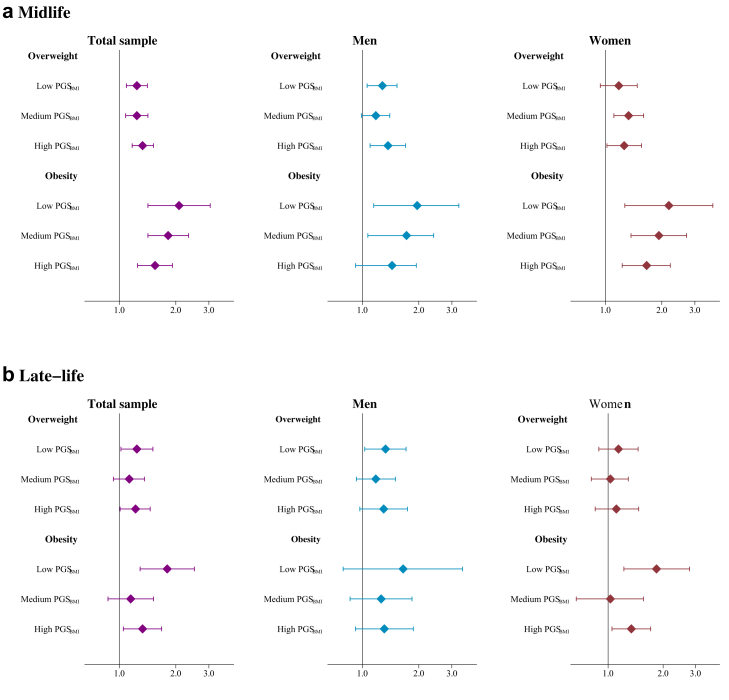
**Interactive effects between BMI category and the PGS**_**BMI**_**.** Hazard rate ratios and 95% confidence intervals of cardiovascular disease in relation to overweight or obesity measured in a) midlife and b) late-life, stratified by genetically predicted low, medium or high BMI. The models are adjusted for study, sex, smoking and education. *BMI* body mass index, *PGS* polygenic score.

There was a statistically significant interaction between obesity and the PGS_BMI_, with 14% lower risk of CVD for individuals with obesity and one SD higher PGS_BMI_, compared to those with obesity and a mean PGS_BMI_ (Table 2). When stratifying by tertiles of the PGS_BMI_, this was seen as a stronger association between obesity and CVD risk among individuals with a genetically predicted low BMI than those with genetically predicted high BMI (Fig. 2a and Supplementary Table S3), ranging from 2.08 times higher risk among those with genetically predicted low BMI to 1.55 times higher risk among those with genetically predicted high BMI. This pattern was consistent in sex-stratified analyses (Fig. 2a and Supplementary Table S3). No interaction was present between overweight and the PGS_BMI_ (Table 2), and no difference in the association between overweight and CVD seen across PGS_BMI_ categories (Fig. 2a and Supplementary Table S3).

#### Co-twin control analyses

**Fig. 3:**
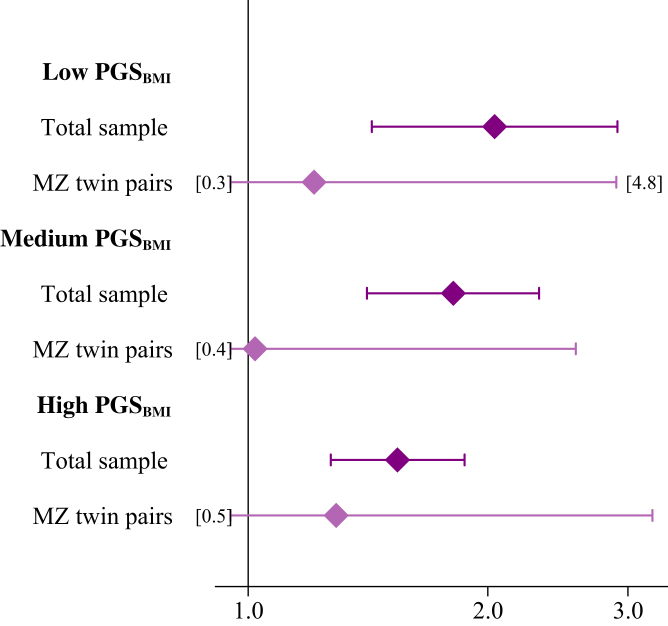
**Co-twin control analyses.** Hazard rate ratios and 95% confidence intervals of cardiovascular disease in relation to obesity measured in midlife, stratified by genetically predicted low, medium or high BMI, for the total sample in dark color and within monozygotic twin pairs in lighter color. The models are adjusted for study, sex, smoking and education, with age as the underlying time scale. *BMI* body mass index, *MZ* monozygotic, *PGS* polygenic score.

The effect estimates of obesity on CVD risk was attenuated in co-twin control analyses compared to the full sample, with a stronger attenuation within monozygotic (HR 1.14, 95% CI 0.64–2.02) than dizygotic (HR 1.64, 95% CI 1.11–2.42) twin pairs. The association between overweight and CVD was similar to that in the total population in dizygotic twin pairs (HR 1.35, 95% CI 1.10–1.66), but attenuated in monozygotic twin pairs (HR 1.08, 95% CI 0.80–1.46). Within dizygotic twin pairs, the joint effect and interaction model results, including the PGS_BMI_, were largely comparable to those of the total sample (Table 2). However, when stratifying monozygotic twin pairs into PGS_BMI_ groups, the difference in the association between obesity and CVD risk between those with a genetically predicted low versus high BMI seen in the total sample disappeared; and the HR was 1.21 for those with a low PGS_BMI_ and 1.29 for those with a high PGS_BMI_ (Fig. 3 and Supplementary Table S4).

### Late life BMI category and the PGS_BMI_ in relation to risk of CVD

#### Independent and joint effect models of BMI category and the PGS_BMI_

In the independent effect models, late life overweight and obesity were associated with a 22% and 40% higher risk of CVD, respectively (Table 2). One SD higher PGS_BMI_ was associated with a 9% higher risk of CVD. In the joint effect model, all results were slightly attenuated although still statistically significant (Table 2). The associations between overweight and CVD as well as of the PGS_BMI_ and CVD were slightly stronger in men than in women, while the effect of obesity was comparable between sexes.

#### Interactive effects between BMI category and the PGS_BMI_

Including an interaction term between late-life obesity and the PGS_BMI_ indicated the same pattern of associations as when BMI was measured in midlife, but of lower power and with a statistically non-significant interaction effect (Table 2). When stratifying by tertiles of the PGS_BMI_, the association between late-life obesity and CVD was also similar to that in midlife, with a higher risk of CVD among those with a genetically predicted low BMI, than those with a genetically predicted high BMI, but with the lowest association among those with a genetically predicted medium BMI (Fig. 2b). The associations ranged between 1.80 times higher risk among those with genetically predicted low BMI to 1.33 times higher risk among those with genetically predicted high BMI (Fig. 2b). The same pattern was seen in sex stratified analyses. There were no differences in the associations between overweight and CVD across PGS_BMI_ groups. Effect estimates and 95% CIs are available in the Supplementary Table S3.

#### Co-twin control analyses

Compared to the total population, the association between late-life BMI category was stronger within dizygotic (HR 1.30, 95% CI 0.97–1.74 for overweight, HR 1.42, 95% 0.92–2.21 for obesity) and monozygotic (HR 1.54, 95% CI 0.79–3.03 for overweight, HR 2.18, 95% 0.68–7.02 for obesity) twin pairs. Similarly, the associations in joint effect and interaction models were generally stronger in co-twin control analyses within dizygotic twin pairs (Table 2). However, it should be noted that the sample size was limited. Stratifying monozygotic twin pairs by genetically predicted low, medium, or high BMI suffered from power issues with very wide confidence intervals, and robust interpretations could not be made (Supplementary Table S4).

### Sensitivity and post-hoc analyses

Results from the competing risk regression, evaluating the risk of incident CVD while considering mortality as the competing event, were consistent with those from the main analyses (Supplementary Table S5). The association between late-life BMI category and the risk of stroke was overall weaker than that for non-stroke CVD (and any CVD), but results were otherwise comparable between non-stroke CVD and stroke (Supplementary Table S6 and S7). Results based on the total analytical sample, regardless of age at BMI measurement, were consistent with the main findings, and closest to those of midlife measured BMI (Table S8).

## Discussion

In this study of genetic influences on the association between BMI and CVD, we examined how genetically predicted BMI interacts with overweight and obesity, in relation to the risk of CVD. Using longitudinal data from the STR, we confirm that both midlife and late-life overweight and obesity as well as the PGS_BMI_ are associated with a higher risk of CVD in independent effect models, with only slight attenuations when mutually adjusted for in joint effect models. Moreover, there was an interaction between midlife obesity and the PGS_BMI_, and when stratifying individuals into genetic predisposition to low, medium, or high BMI, the association between obesity and CVD was stronger with lower genetically predicted BMI. As obesity in the absence of genetically predicted high BMI is then influenced by other factors; i.e. environmental factors such as lifestyle, this indicates differences between environmentally versus genetically influenced obesity, in relation to risk of CVD. Results were similar for the interaction between the PGS_BMI_ and late-life obesity. However, it should be noted that the sample with BMI measured in late life was of limited size, resulting in wide confidence intervals limiting interpretability, and we therefore focus on the midlife sample when interpreting the results from subgroup analyses.

To the best of our knowledge, few studies have explored differences between genetically and environmentally influenced obesity. We first found this difference in relation to dementia where higher BMI in midlife was associated with higher risk of dementia only for those with genetically predicted low BMI,^9^ and later saw the same pattern in relation to cognitive abilities in the Health and Retirement Study.^10^ Additionally, Vinneau et al.^11^ demonstrated the same differences between genetically versus environmentally influenced obesity in relation to mortality in the Health and Retirement Study, showing that older adults with obesity despite a low PGS_BMI_ had the greatest risk of mortality compared to other groups. Recently, Davidson et al.^12^ found the same difference in relation to CVD-related outcomes, also using the Health and Retirement Study, showing that individuals with obesity despite a low PGS_BMI_ have significantly worse health outcomes, including heart problems, compared to their counterparts with genetic obesity. Thus, this difference in associations between genetically versus environmentally influenced obesity has been seen for several different outcomes, and not only in the STR sample (where virtually all twins are born in Sweden to at least one parent also born in Sweden,^27^ limiting generalizability) but also in the more diverse US based Health and Retirement Study, indicating external validity.

Taken together, results from the current and previous studies support the theory that genetically influenced obesity is not associated with negative health outcomes to the same extent as environmentally influenced obesity. Importantly, this indicates that the negative health effects of obesity may be influenced by other factors, rather than by the obesity in itself, as we would otherwise expect similar effects of obesity, regardless of if it is predicted by genetic predisposition or environmental factors. Based on work from animal models, body weight homeostasis is thought to be biologically controlled through neuroendocrine and metabolic pathways. As reviewed by Müller and colleagues,^28^ three theories of biological control of body weight have been developed: 1) the set point model, where body weight is tightly regulated around an inherent set point through feedback loops; 2) the settling point model where a new steady state of body weight can be reached through adaptation to new environments; and 3) the dual-intervention point model which combines the other two by suggesting a range within which body weight can change in response to biological (including genetic and epigenetic) and environmental influences. In the third model, biological influences are controlled through feedback loops, while environmental influences are not, and may override the biological control. In relation to the current study, it is plausible that individuals with a genetically predicted high BMI are better adapted to obesity due to a higher inherent set point or body weight range. In addition, as genetically predicted obesity is inherently biologically influenced it may better maintain body weight homeostasis through feedback loops, compared to environmentally influenced obesity which, according to the dual-intervention point model, is not regulated through feedback loops and may even override biological body weight control.^28^ While warranting further investigation, we could not study differences in environmental influences such as lifestyle, medication, social factors, physical environment or socioeconomic status in the current study, as exposure data were collected at various time points and through various methods, substantially limiting harmonization of such measures.

Further strengthening the hypothesis that obesity in itself does not directly influence CVD risk, the associations were attenuated within twin pairs, with a further attenuation within monozygotic twin pairs. If effect estimates remain stable within twin pairs (especially within monozygotic twin pairs, sharing identical DNA) it indicates, but is not proof of, a causal association. In contrast, if the effect estimate is close to zero within twin pairs, it is strong evidence against a causal association, as it indicates that the association is driven by genetic or other familial confounding.^26^ Our analyses demonstrated an attenuated, but not null association within twin pairs, indicating that at least part of the association is explained by genetic or other familial confounding. This is in line with previous work based on the STR, where the association between BMI and CVD mortality was substantially attenuated in co-twin control models compared to the total sample.^29^ Interestingly, the difference in the association between genetically versus environmentally predicted obesity and CVD was not seen in monozygotic twin pairs. As most traits, including lifestyle behavioral traits, have strong genetic influences,^30^ the lack of a difference between the high and low PGS_BMI_ group among monozygotic twin pairs indicates that the association between obesity and CVD is influenced by additional genetic factors that are not captured by the PGS_BMI_, alternatively by environmental factors shared between the monozygotic twin pairs. This demonstrates the importance and benefits of co-twin control analyses, to fully adjust for genetic or familial influences which cannot be captured by a PGS.

The value of the STR data lies not only in the possibility of conducting co-twin control analyses, but also in its richness and long follow-up. In the current study, we utilized this by including genotyped sub-studies within the STR, all with CVD information from register linkage and BMI from a variety of data collections. While it has been argued that twins are dissimilar from the general population and therefore not representative, previous findings have shown that twins are not significantly different from singletons.^31^ The multiple data collections within the STR limited the number of individuals with missing information, and with the addition of nationwide register information strengthens the representativeness of the study participants. However, some limitations of the data deserves mentioning. First, some BMI measures relies on self-reported measures of height and weight. While self-reported measures can be imprecise, we have shown that the measures are acceptable in the STR data.^32^^,^^33^ Retrospectively self-reported weight comes with substantial individual variability, but in SATSA 82% reported their weight 20 years ago within 10% from their prior measured weight.^32^ Self-reported current height and weight led to very small but increasing differences in height (0.038 cm/year) and BMI (0.016 units/year), but not weight, over time, compared to measured height and weight.^33^ Moreover, the data have been carefully cleaned, comparing longitudinal information about self-reported and measured height and weight.^9^ Nevertheless, there may be some misclassification of BMI category, which, assuming it is non-differential in relation to the outcome, would likely bias the associations towards the null. Disease diagnoses, including CVD categories, from Swedish registers have generally high validity with positive predictive values, around 85–95%.^19^ However, register data are not without limitations. We included the NPR and CDR, thus capturing outpatient specialist care, hospitalizations, and registered causes of death. Primary care is however not included, and milder diagnosis may therefore be missed. Nevertheless, the richness of the data allowed access to BMI information covering a large part of the twins’ lifespan together with objective measures of CVD from register diagnoses for all participants. We could thereby study long-term effects of midlife BMI, the longest follow-up being 62 years, in relation to CVD risk.

The association between midlife obesity and CVD was stronger among women than men, but the associations between overweight or obesity and CVD were present across sex. This is in line with reviews on the topic, which have found that the association between obesity-related traits and CVD is mostly identical between men and women [e.g.^34^, ^35^, ^36^]. Sensitivity analyses comparing stroke versus non-stroke CVD also showed comparable results, indicating that the same underlying mechanisms, driving the association between overweight or obesity and CVD risk, affect both stroke and non-stroke CVD. Competing risk regression, with death as the competing event, showed the same pattern as the main analyses, indicating that the results are not explained by survival bias. These sensitivity analyses strengthen the findings by drawing from the complete follow-up through register linkage. However, studies of older individuals always suffer from bias due to attrition rates and differential selection and survival.^37^ Despite having complete register follow-up, and despite consistent results in competing risk regression, the results may still suffer from such bias. Another potential limitation of the current study is the historical effects of the included cohorts, as e.g. lifestyles and treatment options have changed during the large time span of when the twins were born. This may also explain any differences between the midlife and late-life sample, as environmentally influenced obesity may have a different interpretation in earlier versus later born cohorts. Additionally, pharmacological treatments targeting obesity were not available when BMI was measured in the current study, and as such treatment may affect the association between obesity and CVD this may limit the representativeness of the findings in current times. While we cannot rule out cohort differences affecting the main results, the robustness of the findings within dizygotic twin pairs indicate that cohort differences do not substantially affect our findings, as twins are, by default, always matched on birth year.

In conclusion, while it is important to note that overweight and obesity were associated with a higher risk of CVD across all PGS_BMI_ categories, obesity influenced by environmental factors may be more deleterious than obesity influenced by genetic factors. Interestingly, these differences were not seen when comparing monozygotic twin pairs, indicating that there are genetic or shared environmental factors, not captured by the PGS, influencing the associations.

## Contributors

EO contributed to the literature search, formal analysis, visualization, project administration, and writing the original draft as well as writing review and editing. YZ, JJ, CAR, and AKDA all contributed to the conceptualization, funding acquisition, and to writing review and editing. AKDA also contributed to the data curation. IKK contributed the conceptualization, data curation and formal analysis, funding acquisition, supervision, and writing the original draft as well as writing review and editing. Both EO and IKK directly accessed and verified the underlying data. All authors read and approved the final version of the manuscript.

## Data sharing statement

Data from the STR is an international resource and can be applied for at https://ki.se/en/research/swedish-twin-registry-for-researchers.^14^ The analytical codes and documents (including analysis plan, logs, and output) are available on GitHub: https://github.com/ik-karlsson/Obesity_PRS_CVD.

## Declaration of interests

JJ reports grant funding paid to their institution from the 10.13039/501100004359Swedish Research Council (grant 2018-02077), 10.13039/501100002341Academy of Finland (grant 349335), 10.13039/501100006306Sigrid Jusélius Foundation, 10.13039/100010114Yrjö Jahnsson Foundation, and 10.13039/501100008413Instrumentarium Science Foundation. CAR reports honorarium from the National Institute on Aging and grant funding from the 10.13039/100000002National Institutes of Health (grants NIH 1R01 DA054087, NIH 2R01 AG046938, NIH 2R01 AG050595, NIH 1R01 AG059329, NIH 1RF1 AG058068, NIH 1R01 AG077742, and NIH 1R01 AG053217). IKK reports payments to their institution from the Karolinska Institutet Research Foundation (grant 2022-01718) and Eurolife (mobility grant). IKK also reports payment via a Promising Researcher Award from The Nordic Gerontological Federation. All other authors declare no competing interests.
