# Supplementary material for: Genetically and environmentally predicted obesity in relation to cardiovascular disease: a nationwide cohort study
Source: eClinicalMedicine. 2023 Apr 6;58:101943. doi: 10.1016/j.eclinm.2023.101943 (PMC10166783; doi:10.1016/j.eclinm.2023.101943)
Supplement: Supplemental Material [file mmc1.docx]

**Supplemental material**

Table of contents

[Table S1. Descriptive statistics of the sample with and without genotype data 2](#_Toc129850731)

[Healthcare registers in Sweden 3](#_Toc129850732)

[Table S2. ICD codes used to define cardiovascular disease, divided into non-stroke cardiovascular disease and stroke, and specific codes to define myocardial infarction 3](#_Toc129850733)

[Computation of polygenic score for body mass index 4](#_Toc129850734)

[Figure S1: Distribution of the raw PGS_BMI_ by genotyping collection 5](#_Toc129850735)

[Figure S2: Survival curves visually inspecting proportional hazards 6](#_Toc129850736)

[a. Midlife measures 6](#_Toc129850737)

[b. Late-life measures 7](#_Toc129850738)

[Table S3. Risk of CVD in relation to midlife and late-life BMI category and a polygenic score for BMI 8](#_Toc129850739)

[Table S4. Co-twin control analyses within dizygotic and monozygotic twin pairs of the risk of CVD in relation to midlife and late-life BMI category, stratified by tertiles of the PGS_BMI_ 9](#_Toc129850740)

[Table S5. Competing risk regression of CVD risk in relation to midlife and late-life BMI category and a polygenic score for BMI 10](#_Toc129850741)

[Table S6. Risk of non-stroke CVD in relation to midlife and late-life BMI category and a polygenic score for BMI 12](#_Toc129850742)

[Table S7. Risk of stroke in relation to midlife and late-life BMI category and a polygenic score for BMI 14](#_Toc129850743)

[Table S8. Risk of CVD in relation to BMI category and a polygenic score for BMI, in the total sample and by sex. 16](#_Toc129850744)

[References 17](#_Toc129850745)

# **Table S1. Descriptive statistics of the sample with and without genotype data**

|  | **All** | | **No genotype data** | | **Genotype data** | |
| --- | --- | --- | --- | --- | --- | --- |
|  | 45337 | | 25976 | | 19361 | |
| Birth year, M (SD) | 1940 | (12.16) | 1939 | (12.55 ) | 1942 | (11.25 ) |
| Age at baseline, M (SD) | 59·30 | (11·02) | 60·83 | (11·57) | 57·11 | (9·78) |
| Age at death, M (SD) | 79·43 | (10·67) | 79·20 | (10·72) | 80·00 | (10·50) |
| Low education, N* (%) | 13114 | (28.77) | 8745 | (66.68 ) | 4369 | (33.32 ) |
| Smokers, N** (%) | 27234 | (60.25) | 15737 | (57.78 ) | 11497 | (42.22 ) |

*N = 298 missing for education. **N = 675 missing for smoking. Descriptive statistics for all individuals, individuals without genotype dataand with genotype data (polygenic scores). Statistics are presented as number (%) of individuals for categorical variables and mean level (SD) for continuous variables. *M* mean, *N* number, *SD* standard deviation, *PRS* polygenic risk score.

# Healthcare registers in Sweden

The STR is connected to a number of population-based registries through the 10-digit personal identification number that is assigned to all residents in Sweden. For the current study, information on cardiovascular disease was obtained from both the National Patient Registry (NPR) and the Cause of Death Registry (CDR).

The NPR was started in 1964, and has since 1987 a coverage that contains information about 99% of all in-patient care from hospitals in Sweden (1). For each hospitalization, the primary diagnosis is recorded together with up to 20 additional diagnoses, which is done according to International Classification of Diseases (ICD) codes. The NPR also covers outpatient specialist care since 2001. In addition, all surgeries are recorded (including day surgery) according to surgical codes. The CDR has since 1961 included information about underlying and contributory causes of death for all Swedish residents, which are reported according to ICD codes as well (2). Currently, data from the NPR and CDR are available through the end of 2016. Both primary and additional diagnoses from the NPR was used together with underlying and contributing causes of death from the CDR, as criteria for disease. The ICD codes used to retrieve cardiovascular disease diagnoses are reported in Table S2.

# **Table S2**. ICD codes used to define cardiovascular disease, divided into non-stroke cardiovascular disease and stroke, and specific codes to define myocardial infarction

|  | **ICD-7** | **ICD-8** | **ICD-9** | **ICD-10** | **Surgical code** |
| --- | --- | --- | --- | --- | --- |
| **Non-stroke CVD** | 420 | 410 | 410 | I20 | 984 |
|  | 450 | 411 | 411 | I21 | 3068 |
|  | 453·33 | 412 | 412 | I22 | 3080 |
|  |  | 413 | 413 | I23 | 3127 |
|  |  | 414 | 414 | I24 | 3141 |
|  |  | 440 | 440 | I25 | 3158 |
|  |  | 443·90 | 443X | I79 | FNC |
|  |  |  |  | I73·9 | FND |
|  |  |  |  |  | FNE |
|  |  |  |  |  | FNG00 |
|  |  |  |  |  | FNG02 |
|  |  |  |  |  | FNG05 |
| **Stroke** | 330 | 430 | 430 | I60 |  |
|  | 331·00 | 431 | 431 | I61 |  |
|  | 331·01 | 433 | 434 | I63 |  |
|  | 331·09 | 434 | 436 | I64 |  |
|  | 331·99 | 436 |  |  |  |
|  | 332·00-19 |  |  |  |  |
|  | 332·29 |  |  |  |  |
|  | 334·00-98 |  |  |  |  |

*CVD* cardiovascular disease; *ICD* International Classification of Diseases; *MI* myocardial infarction

# Computation of polygenic score for body mass index

In the current study, genotype information was available from three different collections. As part of TwinGene (3), individuals underwent a health checkup which included collection of blood samples. DNA was extracted and analyzed on the Human OmiExpress array. Out of the 12,630 individuals who underwent a health checkup in TwinGene, genotyping is available for 10,960, analyzed on the Human OmniExpress array. SALT-Y (3) included self-report paper-questionnaire an internet-based investigation, and saliva collection for DNA extraction. Out of the 11,482 participants, 6,403 were genotyped on the Illumina PsychArray. Blood samples were collected as part of the in-person testing phases in SATSA(4) (n=859 participants) and GENDER (5) (n=496 participants). In HARMONY (6), all twins suspected of dementia during the cognitive screening over telephone, together with their co-twins and a control sample of cognitively intact twins, were invited to a clinical workup, which included collection of blood samples (n= 1,557). DNA were extracted, and SATSA, GENDER, and HARMONY were genotyped together on the Illumina PsychArray (n=2,052). The genotype data have all been imputed against the Haplotype Reference Consortium reference panel (7). In the current study, we used SBayesR (8) to compute the polygenic score for BMI (PGS_BMI_). The method uses HapMap3 SNPs, and we therefore selected all HapMap3 SNPs with high quality imputation (INFO score >0.8) and minor allele frequency above 1% in all three genotyping collections, resulting in 952,885 SNPs (out of the ~1.1 million HapMap3 SNPs).

For weights, we used summary statistics from the genome-wide association study (GWAS) of BMI by Yengo and colleagues (9). The GWAS included ~700,000 individuals, combining the largest previous GWAS (age range 12-108) with UK Biobank data (age range 40-69). In follow-up analyses a PGS using SNPs with p<0.001 explained 14% of the variance in BMI (9). The STR samples were included in the GWAS of BMI, and, as any overlap in individuals between the GWAS and the target sample can substantially inflate the predictive power of the PGS(10), we first obtained updated summary statistics where the STR samples had been excluded. To deal with linkage disequilibrium (LD; correlation between nearby SNPs), the summary statistics were processed with SBayesR to obtain shrunken effect sizes, considering the LD pattern (8). The PGS was then computed (separately in each study) using the --score command in Plink2. The --score command computes the PGS by, for each individual, summing up the number of effect alleles at each SNP (0, 1, or 2) across the genome, weighted by the effect size of that SNP in the SBayesR processed GWAS summary statistics. I.e.:

$${PGS}_{i}= \sum_{j}^{N} \beta_{j}*{EA}_{ij}$$

Where β_j_ is the effect size for SNP *j*, and EA_ij_ the number of affect alleles for individual *i* at SNP *j*. Example scripts of the SBayesR processing and computation of the PGS is available on GitHub: <https://github.com/IGEMS/PGS_pipeline>. The distribution of the raw PGS values was very similar across the genotyping collections, as seen in Figure S2.

Principal components (PCs), to adjust for genetic ancestry, were computed on the three genotyping collections combined, using a strict set of SNPs with very high genotyping quality (info score >0.95) and minor allele frequency >5% in all three data collections. As an option to controlling for PCs in all analytical models, the PCs can be regressed out from the PGS prior to analyses, thus obtaining a PC-adjusted PGS. In the current study, such a simplified approach was desirable as the tertiles of the PGS can then be defined based on a PGS already accounted for PCs. To test that models with the PC-adjusted PGS are comparable to models of the original PGS and PCs as covariates, we first compared the results between such models in linear regression with BMI as the outcome (using the midlife measure of BMI if available, and otherwise the late-life measure). To obtain a PC-adjusted PGS we regressed out the first five PCs using a linear regression model, and used the residuals as the PC-adjusted PGS. Both PGS were standardized prior to analyses. The models were adjusted for age at BMI measure, sex, and study (genotyping collection), and models of the original PGS additionally for the first five PCs. Variance explained by the PGS was calculated as the R^2^ from the model minus the R^2^ from a model with all covariates but without the PGS. Both the estimate from the linear model and the variance explained was near-identical for the PC-adjusted PGS (β=1.110, SE=0.026, p<0.01; R^2^=0.1085) and the original PGS adjusted for PCs (β=1.117, SE=0.027, p<0.01; R^2^=0.1086). Therefore, we used the PC-adjusted PGS in the current study, standardized within each genotyping collection sample, to mean=0 and standard deviation=1.

# Figure S1: Distribution of the raw PGS_BMI_ by genotyping collection


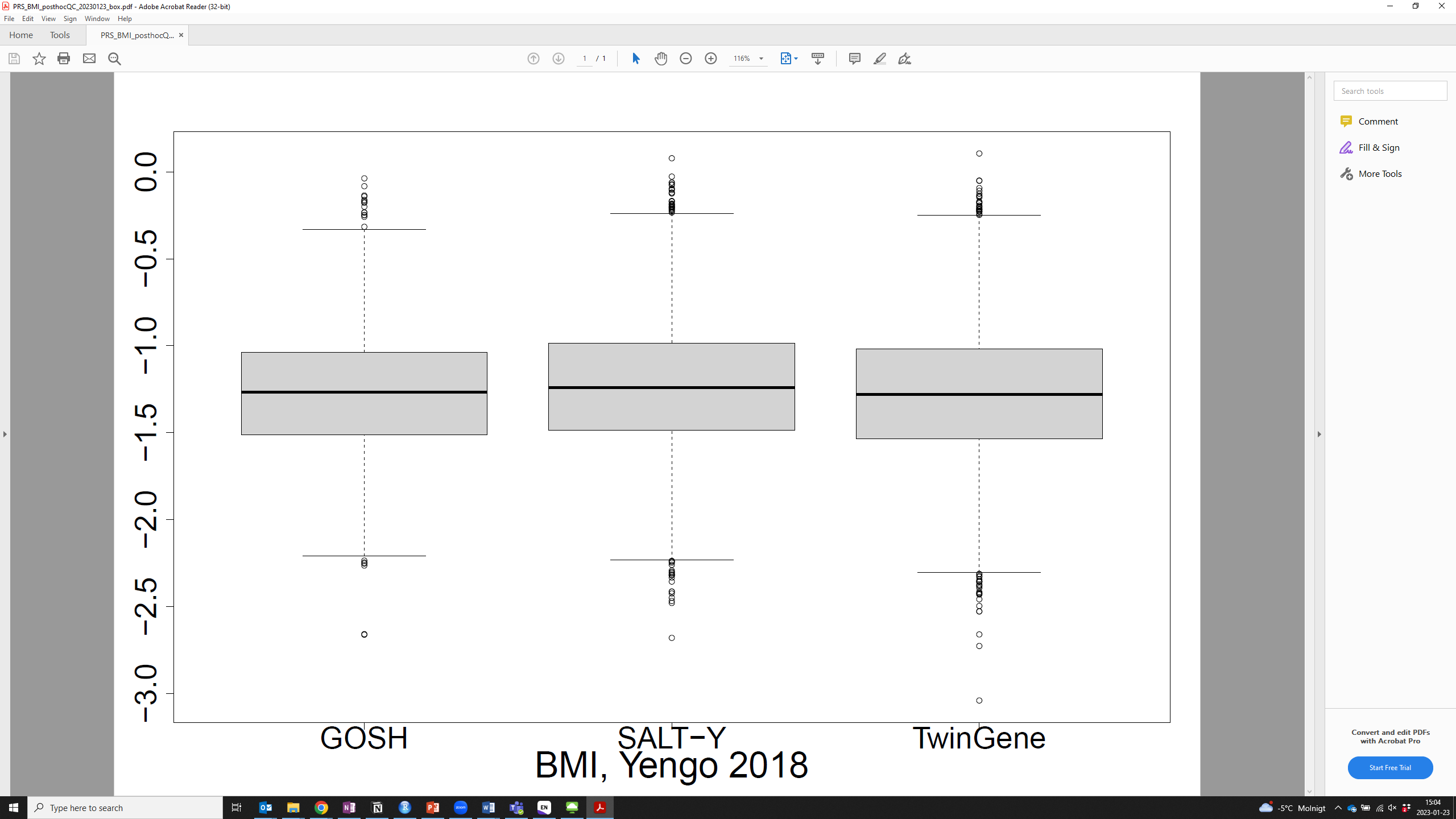


Testing multicollinearity

To assess potential issues of collinearity between BMI and the PGS_BMI_, we first examined the correlation matrix from the joint effect model described above, but with BMI as a continuous measure, resulting in correlations of -0.35 for midlife and -0.36 for late-life measures of BMI. In addition, we assessed the variance inflation factor (VIF) from a linear regression model (as the VIF does not translate to Cox models) with only BMI and the PGS_BMI_ as predictors of CVD, showing VIF of 1.12 for the midlife sample and 1.11 for the late-life sample. To assess multicollinearity across covariates, we added sex, education, and smoking to the linear model, which resulted in a mean VIF of 1.07 for both the midlife and late-life sample. In the latter model, the VIF for BMI and the PGS_BMI_ were the highest, at 1.16 and 1.14, respectively, in midlife and 1.11 for both in late-life. Thus, neither correlations nor VIF values indicate issues with multicollinearity.

# Figure S2: Survival curves visually inspecting proportional hazards

# Midlife measures


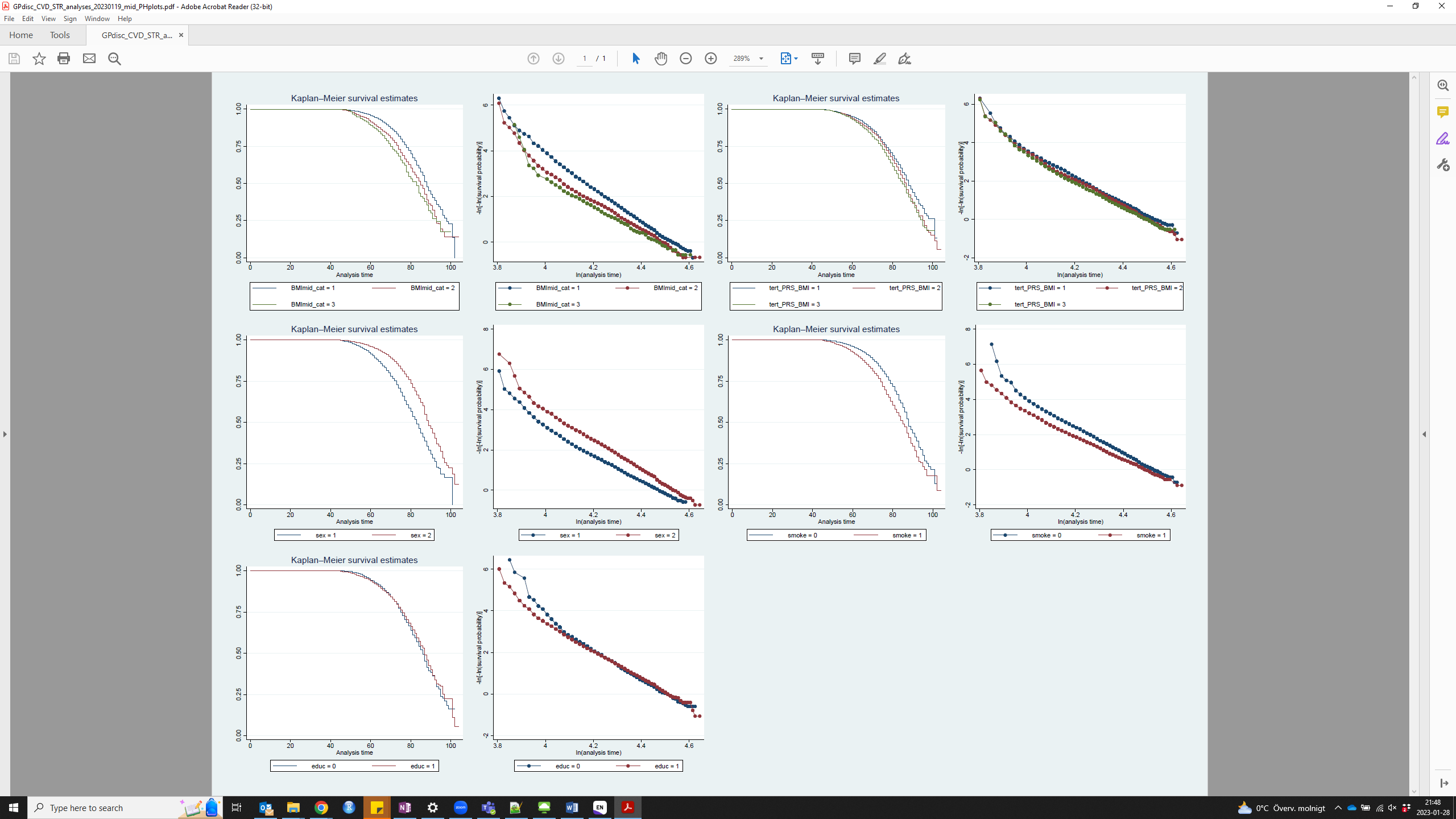


# Late-life measures


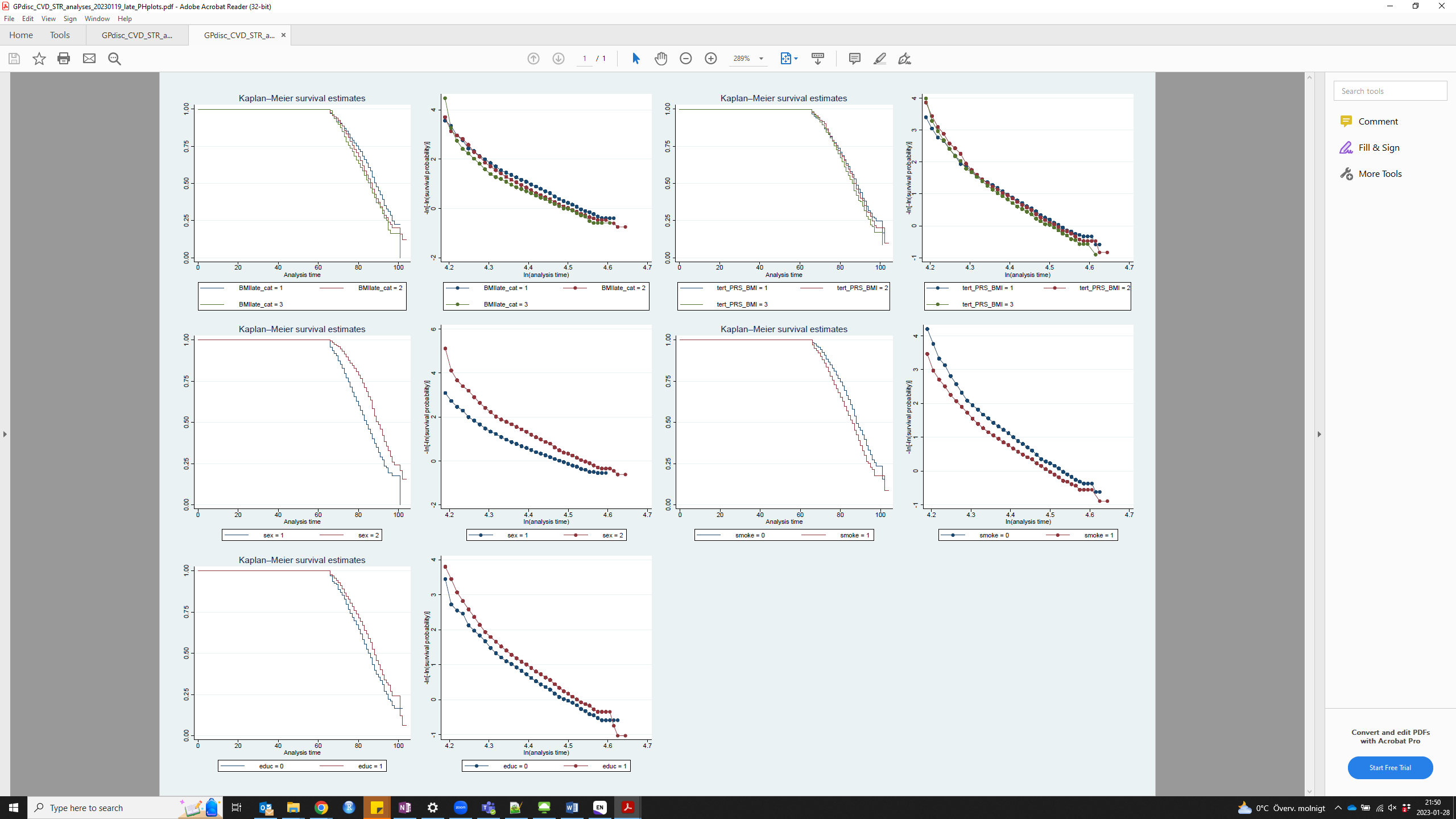


# **Table S3**. Risk of CVD in relation to midlife and late-life BMI category and a polygenic score for BMI

| **a) Midlife** | **All** | **Men** | **Women** |
| --- | --- | --- | --- |
| **By tertiles of the PGS_BMI_** |  |  |  |
| Overweight, low PGS_BMI_ | **1.24 (1.09-1.41), p=0.001** | **1.28 (1.06-1.53), p=0.009** | 1.18 (0.94-1.48), p=0.163 |
| Overweight, medium PGS_BMI_ | **1.24 (1.08-1.42), p=0.002** | 1.18 (0.99-1.40), p=0.060 | **1.33 (1.11-1.60), p=0.002** |
| Overweight, high PGS_BMI_ | **1.33 (1.17-1.52), p<0.001** | **1.37 (1.10-1.70), p<0.001** | **1.26 (1.02-1.56), p<0.001** |
| Obesity, low PGS_BMI_ | **2.08 (1.42-3.05), p<0.001** | **1.96 (1.17-3.27), p=0.010** | **2.18 (1.27-3.74), p=0.005** |
| Obesity, medium PGS_BMI_ | **1.82 (1.42-2.34), p<0.001** | **1.72 (1.23-2.40), p=0.002** | **1.93 (1.37-2.71), p<0.001** |
| Obesity, high PGS_BMI_ | **1.55 (1.25-1.92), p<0.001** | **1.44 (1.07-1.94), p=0.015** | **1.66 (1.23-2.22), p=0.001** |
| **b) Late-life** | **All** | **Men** | **Women** |
| **By tertiles of the PGS_BMI_** |  |  |  |
| Overweight, low PGS_BMI_ | **1.24 (1.02-1.51), p=0.035** | **1.33 (1.03-1.71), p=0.027** | 1.14 (0.89-1.46), p=0.300 |
| Overweight, medium PGS_BMI_ | 1.13 (0.93-1.36), p=0.209 | 1.18 (0.93-1.50), p=0.172 | 1.02 (0.81-1.29), p=0.859 |
| Overweight, high PGS_BMI_ | **1.22 (1.01-1.46), p=0.038** | 1.30 (0.97-1.74), p=0.038 | 1.11 (0.85-1.47), p=0.038 |
| Obesity, low PGS_BMI_ | **1.80 (1.29-2.51), p=0.001** | **1.65 (0.79-3.42), p=0.180** | **1.84 (1.22-2.79), p=0.004** |
| Obesity, medium PGS_BMI_ | 1.15 (0.87-1.52), p=0.336 | 1.26 (0.86-1.84), p=0.240 | 1.03 (0.67-1.56), p=0.902 |
| Obesity, high PGS_BMI_ | **1.33 (1.05-1.68), p=0.018** | 1.31 (0.92-1.87), p=0.134 | **1.34 (1.05-1.71), p=0.020** |

Hazard rate ratios (95% confidence intervals) of CVD in relation to midlife or late-life BMI category, stratified by genetically predicted low, medium or high BMI. All models are adjusted for study, sex, smoking and education, and age used as the underlying time scale. An interaction term was also used to stratify the association between BMI category and non-stroke CVD risk by tertiles of the PGS_BMI_. Statistically significant estimates (at the α<0.05 level) are presented in bold. *BMI* body mass index, *CVD* cardiovascular disease, *PGS* polygenic score.

# **Table S4.** Co-twin control analyses within dizygotic and monozygotic twin pairs of the risk of CVD in relation to midlife and late-life BMI category, stratified by tertiles of the PGS_BMI_

| **a)** **Midlife** | **Dizygotic twin pairs** | **Monozygotic twin pairs** |
| --- | --- | --- |
| **By tertiles of the PGS_BMI_** |  |  |
| Overweight, low PGS_BMI_ | **1.45 (1.00-2.09), p=0.049** | 0·94 (0·53-1·66), p=0·827 |
| Overweight, medium PGS_BMI_ | **1.46 (1.02-2.08), p=0.037** | 1·15 (0·73-1·82), p=0·548 |
| Overweight, high PGS_BMI_ | 1.12 (0.79-1.58), p=0.523 | 1·14 (0·64-2·05), p=0·656 |
| Obesity, low PGS_BMI_ | 2.30 (0.69-7.70), p=0.177 | 1·21 (0·30-4·83), p=0·791 |
| Obesity, medium PGS_BMI_ | 1.55 (0.76-3.16), p=0.231 | 1·02 (0·40-2·58), p=0·965 |
| Obesity, high PGS_BMI_ | 1.36 (0.81-2.28), p=0.239 | 1·29 (0·51-3·22), p=0·591 |
| **b) Late-life** | **Dizygotic twin pairs** | **Monozygotic twin pairs** |
| Overweight, low PGS_BMI_ | 1.51 (0.94-2.42), p=0.089 | 1·00 (0·29-3·46), p=0·997 |
| Overweight, medium PGS_BMI_ | 0.92 (0.56-1.50), p=0.733 | 2·30 (0·86-6·20), p=0·098 |
| Overweight, high PGS_BMI_ | 1.37 (0.78-2.40), p=0.278 | 1·08 (0·22-5·21), p=0·925 |
| Obesity, low PGS_BMI_ | **3.38 (1.17-9.79), p=0.025** | 2·97 (0·22-40·39), p=0·413 |
| Obesity, medium PGS_BMI_ | 0.87 (0.40-1.90), p=0.719 | 1·63 (0·33-8·03), p=0·550 |
| Obesity, high PGS_BMI_ | 1.24 (0.63-2.44), p=0.528 | 4·08 (0·23-70·85), p=0·335 |

Within twin pair hazard rate ratios (95% confidence intervals) of CVD in relation to midlife or late-life BMI category, stratified by genetically predicted low, medium or high BMI. Co-twin control models were applied separately in dizygotic and monozygotic twin pairs. In dizygotic twin pairs, stratification was achieved by including an interaction term between BMI category and the PGS_BMI_ in the model. As co-twin control models relies on pair differences, this results in only twin differing in PGS_BMI_ category, phenotypic BMI category, and the outcome contribute to the interaction effect estimate (although all can contribute to main and covariate effects). In monozygotic twin pairs, stratification was achieved by separately analyzing the three PGS_BMI_ groups. All models are adjusted for sex, smoking and education, and age used as the underlying time scale. Statistically significant estimates (at the α<0.05 level) are presented in bold. *BMI* body mass index, *CVD* cardiovascular disease, *PGS* polygenic score.

# **Table S5.** Competing risk regression of CVD risk in relation to midlife and late-life BMI category and a polygenic score for BMI

| **a)** **Midlife** | **All** | **Men** | **Women** |
| --- | --- | --- | --- |
| **Independent effect model** |  |  |  |
| Overweight | **1·30 (1·20-1·40), p<0·001** | **1·29 (1·17-1·42), p<0·001** | **1·27 (1·12-1·43), p<0·001** |
| Obesity | **1·66 (1·45-1·90), p<0·001** | **1·51 (1·25-1·83), p<0·001** | **1·85 (1·53-2·24), p<0·001** |
| PGS_BMI_ | **1·11 (1·07-1·15), p<0·001** | **1·11 (1·06-1·16), p<0·001** | **1·11 (1·05-1·17), p<0·001** |
| **Joint effect model** |  |  |  |
| Overweight | **1·26 (1·17-1·36), p<0·001** | **1·25 (1·13-1·38), p<0·001** | **1·24 (1·10-1·40), p<0·001** |
| Obesity | **1·57 (1·36-1·80), p<0·001** | **1·41 (1·16-1·73), p<0·001** | **1·76 (1·45-2·14), p<0·001** |
| PGS_BMI_ | **1·06 (1·02-1·10), p=0·003** | **1·06 (1·01-1·12), p=0·016** | 1·05 (0·99-1·12), p=0·075 |
| **Interaction model** |  |  |  |
| Overweight | **1·25 (1·16-1·35), p<0·001** | **1·25 (1·13-1·38), p<0·001** | **1·23 (1·09-1·39), p<0·001** |
| Obesity | **1·72 (1·47-2·02), p<0·001** | **1·54 (1·23-1·93), p<0·001** | **1·95 (1·57-2·44), p<0·001** |
| PGS_BMI_ | **1·07 (1·02-1·13), p=0·011** | 1·07 (1·00-1·15), p=0·060 | 1·07 (0·99-1·15), p=0·081 |
| Overweight * PGS_BMI_ | 1·00 (0·93-1·09), p=0·911 | 1·01 (0·91-1·11), p=0·915 | 1·01 (0·89-1·14), p=0·896 |
| Obesity * PGS_BMI_ | 0·87 (0·75-1·00), p=0·053 | 0·88 (0·72-1·08), p=0·220 | **0·86 (0·71-1·04), p=0·112** |
| **By tertiles of the PGS BMI** |  |  |  |
| Overweight, low PGS | **1·24 (1·08-1·43), p=0·003** | **1·28 (1·07-1·52), p=0·007** | 1·14 (0·90-1·44), p=0·284 |
| Overweight, medium PGS | **1·23 (1·08-1·39), p=0·002** | 1·16 (0·99-1·36), p=0·072 | **1·33 (1·08-1·63), p=0·006** |
| Overweight, high PGS | **1·32 (1·16-1·50), p<0·001** | **1·34 (1·13-1·60), p<0·001** | **1·24 (1·01-1·51), p=0·035** |
| Obesity, low PGS | **1·97 (1·37-2·84), p<0·001** | **1·80 (1·08-3·00), p=0·025** | **2·21 (1·34-3·66), p=0·002** |
| Obesity, medium PGS | **1·71 (1·35-2·18), p<0·001** | **1·57 (1·13-2·19), p=0·008** | **1·87 (1·32-2·65), p<0·001** |
| Obesity, high PGS | **1·48 (1·22-1·80), p<0·001** | **1·34 (1·01-1·77), p=0·040** | **1·67 (1·28-2·17), p<0·001** |

| **b) Late life** | **All** | **Men** | **Women** |
| --- | --- | --- | --- |
| **Independent effect model** |  |  |  |
| Overweight | **1·19 (1·07-1·32), p<0·001** | **1·27 (1·10-1·46), p<0·001** | 1·09 (0·93-1·27), p=0·292 |
| Obesity | **1·33 (1·15-1·55), p<0·001** | **1·37 (1·10-1·71), p=0·006** | **1·32 (1·08-1·61), p=0·007** |
| PGS_BMI_ | **1·07 (1·01-1·12), p=0·012** | **1·09 (1·02-1·17), p=0·012** | 1·04 (0·97-1·11), p=0·299 |
| **Joint effect models** |  |  |  |
| Overweight | **1·17 (1·05-1·30), p=0·003** | **1·24 (1·07-1·43), p=0·004** | 1·08 (0·93-1·26), p=0·324 |
| Obesity | **1·29 (1·10-1·51), p<0·001** | **1·30 (1·03-1·64), p=0·029** | **1·31 (1·06-1·62), p=0·013** |
| PGS_BMI_ | 1·04 (0·99-1·09), p=0·150 | 1·07 (0·99-1·15), p=0·090 | 1·01 (0·94-1·09), p=0·761 |
| **Interaction model** |  |  |  |
| Overweight | **1·17 (1·05-1·30), p=0·005** | **1·23 (1·06-1·43), p=0·005** | 1·08 (0·92-1·26), p=0·342 |
| Obesity | **1·35 (1·14-1·60), p<0·001** | **1·42 (1·11-1·82), p=0·006** | **1·32 (1·05-1·66), p=0·019** |
| PGS_BMI_ | 1·05 (0·97-1·14), p=0·242 | 1·07 (0·95-1·20), p=0·250 | 1·02 (0·92-1·14), p=0·671 |
| Overweight * PGS_BMI_ | 1·01 (0·90-1·12), p=0·914 | 1·02 (0·88-1·19), p=0·789 | 0·98 (0·84-1·15), p=0·835 |
| Obesity * PGS_BMI_ | 0·91 (0·78-1·07), p=0·248 | 0·85 (0·66-1·08), p=0·184 | 0·97 (0·79-1·19), p=0·754 |
| **By tertiles of the PGS BMI** |  |  |  |
| Overweight, low PGS | **1·21 (1·01-1·45), p=0·034** | **1·29 (1·01-1·65), p=0·045** | 1·14 (0·88-1·48), p=0·332 |
| Overweight, medium PGS | 1·06 (0·89-1·27), p=0·507 | 1·06 (0·84-1·35), p=0·615 | 1·03 (0·80-1·35), p=0·798 |
| Overweight, high PGS | **1·26 (1·03-1·54), p=0·023** | **1·41 (1·06-1·86), p=0·016** | 1·08 (0·81-1·43), p=0·590 |
| Obesity, low PGS | **1·73 (1·23-2·42), p<0·001** | **1·82 (1·06-3·12), p=0·030** | **1·64 (1·06-2·55), p=0·026** |
| Obesity, medium PGS | 1·10 (0·82-1·46), p=0·525 | 1·21 (0·81-1·82), p=0·354 | 1·02 (0·68-1·52), p=0·933 |
| Obesity, high PGS | **1·32 (1·05-1·67), p=0·019** | 1·30 (0·92-1·85), p=0·143 | **1·38 (1·01-1·87), p=0·043** |

Cause-specific hazard rate ratios (95% confidence intervals) of CVD in relation to midlife or late-life BMI category and PGS_BMI_. CVD was treated as the cause-specific outcome and death as the competing event. All models are adjusted for study, sex, smoking and education, and age used as the underlying time scale. Independent effect models contain either BMI category *or* PGS_BMI_ as predictors of CVD. Joint effect models contain BMI category *and* PGS_BMI_ together as predictors of CVD. Interaction models contain main effects of BMI category and the PGS_BMI_, and an interaction term between BMI category and the PGS_BMI_. An interaction term was also used to stratify the association between BMI category and non-stroke CVD risk by tertiles of the PGS_BMI_. Statistically significant estimates (at the α<0.05 level) are presented in bold. *BMI* body mass index, *CVD* cardiovascular disease, *PGS* polygenic score.

# **Table S6**. Risk of non-stroke CVD in relation to midlife and late-life BMI category and a polygenic score for BMI

| **a)** **Midlife** | **All** | **Men** | **Women** |
| --- | --- | --- | --- |
| **Independent effect model** |  |  |  |
| Overweight | **1·32 (1·21-1·44), p<0·001** | **1·34 (1·19-1·51), p<0·001** | **1·27 (1·12-1·45), p<0·001** |
| Obesity | **1·69 (1·41-2·01), p<0·001** | **1·62 (1·30-2·04), p<0·001** | **1·74 (1·35-2·23), p<0·001** |
| PGS_BMI_ | **1·12 (1·07-1·16), p<0·001** | **1·11 (1·05-1·17), p<0·001** | **1·13 (1·05-1·21), p=0·001** |
| **Joint effect model** |  |  |  |
| Overweight | **1·28 (1·17-1·40), p<0·001** | **1·30 (1·15-1·47), p<0·001** | **1·23 (1·07-1·41), p=0·003** |
| Obesity | **1·58 (1·32-1·89), p<0·001** | **1·53 (1·21-1·94), p<0·001** | **1·61 (1·23-2·10), p<0·001** |
| PGS_BMI_ | **1·07 (1·02-1·11), p=0·002** | **1·06 (1·00-1·12), p=0·042** | **1·08 (1·01-1·16), p=0·035** |
| **Interaction model** |  |  |  |
| Overweight | **1·28 (1·17-1·39), p<0·001** | **1·30 (1·15-1·47), p<0·001** | **1·22 (1·06-1·41), p=0·006** |
| Obesity | **1·68 (1·36-2·07), p<0·001** | **1·68 (1·31-2·14), p<0·001** | **1·65 (1·23-2·22), p=0·001** |
| PGS_BMI_ | **1·08 (1·02-1·14), p=0·006** | 1·08 (0·98-1·19), p=0·116 | 1·08 (0·98-1·18), p=0·104 |
| Overweight * PGS_BMI_ | 1·00 (0·92-1·08), p=0·927 | 0·98 (0·86-1·12), p=0·797 | 1·02 (0·87-1·19), p=0·825 |
| Obesity * PGS_BMI_ | 0·91 (0·78-1·07), p=0·272 | 0·86 (0·68-1·09), p=0·202 | 0·97 (0·77-1·23), p=0·806 |
| **By tertiles of the PGS BMI** |  |  |  |
| Overweight, low PGS | **1·20 (1·04-1·40), p=0·014** | **1·28 (1·06-1·54), p=0·011** | 1·06 (0·80-1·41), p=0·684 |
| Overweight, medium PGS | **1·30 (1·12-1·51), p=0·001** | **1·29 (1·05-1·58), p=0·014** | **1·30 (1·05-1·61), p=0·016** |
| Overweight, high PGS | **1·33 (1·14-1·55), p<0·001** | **1·33 (1·05-1·70), p<0·001** | **1·30 (1·04-1·62), p<0·001** |
| Obesity, low PGS | **1·84 (1·19-2·86), p=0·006** | **1·77 (1·02-3·06), p=0·041** | 1·90 (0·97-3·71), p=0·061 |
| Obesity, medium PGS | **1·66 (1·23-2·24), p=0·001** | **1·78 (1·21-2·62), p=0·003** | 1·50 (0·99-2·27), p=0·056 |
| Obesity, high PGS | **1·55 (1·23-1·94), p<0·001** | 1·41 (0·99-2·00), p=0·055 | **1·69 (1·18-2·41), p=0·004** |

| **b) Late life** |  |  |  |
| --- | --- | --- | --- |
| **Independent effect model** |  |  |  |
| Overweight | **1·22 (1·07-1·40), p=0·003** | **1·40 (1·17-1·67), p<0·001** | 1·02 (0·86-1·21), p=0·844 |
| Obesity | **1·40 (1·17-1·68), p<0·001** | **1·34 (1·02-1·78), p=0·039** | **1·41 (1·13-1·76), p=0·002** |
| PGS_BMI_ | **1·08 (1·02-1·14), p=0·013** | **1·11 (1·03-1·18), p=0·004** | 1·05 (0·97-1·13), p=0·234 |
| **Joint effect models** |  |  |  |
| Overweight | **1·20 (1·04-1·38), p=0·010** | **1·36 (1·14-1·63), p=0·001** | 1·02 (0·86-1·21), p=0·840 |
| Obesity | **1·33 (1·11-1·59), p=0·002** | 1·26 (0·94-1·68), p=0·116 | **1·38 (1·08-1·77), p=0·009** |
| PGS_BMI_ | 1·05 (0·99-1·11), p=0·125 | **1·08 (1·00-1·16), p=0·044** | 1·02 (0·94-1·10), p=0·685 |
| **Interaction model** |  |  |  |
| Overweight | **1·20 (1·04-1·38), p=0·010** | **1·35 (1·13-1·61), p=0·001** | 1·01 (0·85-1·21), p=0·884 |
| Obesity | **1·40 (1·16-1·70), p<0·001** | **1·38 (1·01-1·90), p=0·045** | **1·39 (1·08-1·81), p=0·011** |
| PGS_BMI_ | 1·06 (0·97-1·16), p=0·175 | 1·12 (0·99-1·26), p=0·066 | 1·01 (0·89-1·14), p=0·919 |
| Overweight * PGS_BMI_ | 0·99 (0·88-1·12), p=0·916 | 0·97 (0·82-1·14), p=0·672 | 1·02 (0·86-1·21), p=0·783 |
| Obesity * PGS_BMI_ | 0·91 (0·77-1·08), p=0·274 | 0·78 (0·57-1·07), p=0·130 | 1·01 (0·82-1·23), p=0·953 |
| **By tertiles of the PGS BMI** |  |  |  |
| Overweight, low PGS | 1·23 (0·99-1·54), p=0·065 | **1·48 (1·10-1·98), p=0·009** | 0·98 (0·75-1·28), p=0·905 |
| Overweight, medium PGS | 1·13 (0·92-1·40), p=0·241 | 1·22 (0·90-1·64), p=0·195 | 0·99 (0·75-1·29), p=0·922 |
| Overweight, high PGS | **1·25 (1·00-1·55), p=0·050** | **1·39 (1·00-1·94), p=0·050** | 1·06 (0·76-1·49), p=0·050 |
| Obesity, low PGS | **1·83 (1·24-2·71), p=0·003** | 1·93 (0·91-4·11), p=0·087 | **1·70 (1·00-2·89), p=0·048** |
| Obesity, medium PGS | 1·09 (0·76-1·56), p=0·623 | 1·11 (0·68-1·82), p=0·681 | 1·04 (0·62-1·74), p=0·877 |
| Obesity, high PGS | **1·38 (1·05-1·81), p=0·021** | 1·19 (0·77-1·85), p=0·427 | **1·53 (1·13-2·08), p=0·006** |

Hazard rate ratios (95% confidence intervals) of non-stroke CVD in relation to midlife or late-life BMI category and PGS_BMI_. All models are adjusted for study, sex, smoking and education, and age used as the underlying time scale. Independent effect models contain either overweight, obesity *or* PGS_BMI_ as predictors of non-stroke CVD. Joint effect models contain overweight, obesity *and* PGS_BMI_ together as predictors of non-stroke CVD. Interaction models contain main effects of BMI category and the PGS_BMI_, and an interaction term between BMI category and the PGS_BMI_. An interaction term was also used to stratify the association between BMI category and non-stroke CVD risk by tertiles of the PGS_BMI_. Statistically significant estimates (at the α<0.05 level) are presented in bold. *BMI* body mass index, *CVD* cardiovascular disease, *PGS* polygenic score.

# **Table S7**. Risk of stroke in relation to midlife and late-life BMI category and a polygenic score for BMI

| **a)** **Midlife** | **All** | **Men** | **Women** |
| --- | --- | --- | --- |
| **Independent effect model** |  |  |  |
| Overweight | **1·26 (1·11-1·42), p<0·001** | **1·26 (1·09-1·46), p=0·002** | **1·24 (1·02-1·52), p=0·031** |
| Obesity | **1·70 (1·35-2·14), p<0·001** | **1·49 (1·11-1·99), p=0·008** | **1·90 (1·41-2·56), p<0·001** |
| PGS_BMI_ | **1·11 (1·05-1·17), p<0·001** | **1·11 (1·02-1·20), p=0·010** | **1·11 (1·01-1·21), p=0·032** |
| **Joint effect model** |  |  |  |
| Overweight | **1·22 (1·08-1·39), p=0·001** | **1·21 (1·04-1·42), p=0·013** | 1·22 (0·99-1·50), p=0·058 |
| Obesity | **1·60 (1·26-2·03), p<0·001** | **1·38 (1·01-1·88), p=0·041** | **1·81 (1·30-2·52), p<0·001** |
| PGS_BMI_ | 1·06 (1·00-1·13), p=0·043 | 1·07 (0·98-1·17), p=0·109 | 1·05 (0·95-1·16), p=0·354 |
| **Interaction model** |  |  |  |
| Overweight | **1·21 (1·06-1·37), p=0·004** | **1·21 (1·03-1·42), p=0·021** | 1·20 (0·96-1·49), p=0·106 |
| Obesity | **1·88 (1·43-2·46), p<0·001** | **1·55 (1·11-2·18), p=0·011** | **2·19 (1·52-3·17), p<0·001** |
| PGS_BMI_ | 1·05 (0·97-1·14), p=0·203 | 1·04 (0·92-1·17), p=0·517 | 1·07 (0·94-1·23), p=0·308 |
| Overweight * PGS_BMI_ | 1·07 (0·94-1·22), p=0·293 | 1·10 (0·92-1·30), p=0·291 | 1·04 (0·83-1·29), p=0·750 |
| Obesity * PGS_BMI_ | 0·81 (0·64-1·02), p=0·074 | 0·89 (0·63-1·24), p=0·485 | 0·74 (0·55-1·01), p=0·056 |
| **By tertiles of the PGS BMI** |  |  |  |
| Overweight, low PGS | 1·20 (0·95-1·52), p=0·118 | 1·18 (0·89-1·57), p=0·243 | 1·21 (0·78-1·87), p=0·403 |
| Overweight, medium PGS | 1·16 (0·94-1·42), p=0·171 | 1·16 (0·88-1·51), p=0·296 | 1·20 (0·86-1·66), p=0·283 |
| Overweight, high PGS | **1·31 (1·07-1·61), p=0·008** | 1·34 (0·99-1·82), p=0·008 | 1·23 (0·90-1·69), p=0·008 |
| Obesity, low PGS | **2·14 (1·16-3·94), p=0·015** | 2·16 (0·93-4·98), p=0·072 | 2·07 (0·73-5·83), p=0·169 |
| Obesity, medium PGS | **1·90 (1·29-2·80), p=0·001** | 1·48 (0·89-2·45), p=0·129 | **2·29 (1·31-4·01), p=0·004** |
| Obesity, high PGS | **1·45 (1·07-1·96), p=0·018** | 1·31 (0·80-2·12), p=0·280 | **1·56 (1·05-2·31), p=0·027** |

| **b) Late life** |  |  |  |
| --- | --- | --- | --- |
| **Independent effect model** |  |  |  |
| Overweight | 1·14 (0·99-1·32), p=0·074 | 1·11 (0·92-1·34), p=0·294 | 1·17 (0·92-1·48), p=0·202 |
| Obesity | **1·28 (1·03-1·61), p=0·027** | 1·29 (0·95-1·76), p=0·102 | 1·27 (0·95-1·70), p=0·105 |
| PGS_BMI_ | **1·11 (1·04-1·19), p=0·002** | **1·11 (1·02-1·20), p=0·019** | **1·12 (1·01-1·23), p=0·031** |
| **Joint effect models** |  |  |  |
| Overweight | 1·09 (0·94-1·27), p=0·237 | 1·07 (0·88-1·30), p=0·522 | 1·13 (0·89-1·45), p=0·322 |
| Obesity | 1·19 (0·94-1·50), p=0·146 | 1·21 (0·87-1·67), p=0·262 | 1·18 (0·86-1·61), p=0·306 |
| PGS_BMI_ | **1·09 (1·02-1·17), p=0·017** | 1·09 (0·99-1·19), p=0·077 | 1·10 (0·99-1·22), p=0·090 |
| **Interaction model** |  |  |  |
| Overweight | 1·09 (0·94-1·26), p=0·244 | 1·07 (0·88-1·30), p=0·520 | 1·11 (0·87-1·41), p=0·394 |
| Obesity | 1·26 (0·98-1·62), p=0·069 | 1·18 (0·81-1·70), p=0·392 | 1·32 (0·96-1·81), p=0·083 |
| PGS_BMI_ | **1·16 (1·03-1·31), p=0·017** | 1·11 (0·97-1·28), p=0·125 | **1·21 (1·04-1·41), p=0·016** |
| Overweight * PGS_BMI_ | 0·93 (0·78-1·11), p=0·419 | 0·95 (0·77-1·18), p=0·651 | 0·91 (0·73-1·14), p=0·423 |
| Obesity * PGS_BMI_ | 0·84 (0·65-1·08), p=0·164 | 1·01 (0·72-1·42), p=0·973 | 0·71 (0·50-1·01), p=0·060 |
| **By tertiles of the PGS BMI** |  |  |  |
| Overweight, low PGS | 1·21 (0·91-1·60), p=0·193 | 1·17 (0·85-1·62), p=0·329 | 1·23 (0·83-1·83), p=0·309 |
| Overweight, medium PGS | 1·07 (0·84-1·36), p=0·598 | 1·06 (0·75-1·49), p=0·754 | 1·05 (0·68-1·61), p=0·840 |
| Overweight, high PGS | 1·04 (0·79-1·39), p=0·764 | 1·01 (0·69-1·47), p=0·764 | 1·11 (0·73-1·69), p=0·764 |
| Obesity, low PGS | 1·56 (0·90-2·70), p=0·116 | 0·83 (0·00-3896·69), p=0·9 | **2·22 (1·21-4·06), p=0·010** |
| Obesity, medium PGS | 1·14 (0·77-1·67), p=0·516 | 1·41 (0·81-2·46), p=0·229 | 0·83 (0·42-1·64), p=0·598 |
| Obesity, high PGS | 1·12 (0·82-1·53), p=0·461 | 1·16 (0·71-1·90), p=0·556 | 1·09 (0·67-1·76), p=0·727 |

Hazard rate ratios (95% confidence intervals) of stroke in relation to midlife or late-life BMI category and PGS_BMI_. All models are adjusted for study, sex, smoking and education, and age used as the underlying time scale. Independent effect models contain either overweight, obesity *or* PGS_BMI_ as predictors of stroke. Joint effect models contain overweight, obesity *and* PGS_BMI_ together as predictors of stroke. Interaction models contain main effects of BMI category and the PGS_BMI_, and an interaction term between BMI category and the PGS_BMI_. An interaction term was also used to stratify the association between BMI category and stroke risk by tertiles of the PGS_BMI_. Statistically significant estimates (at the α<0.05 level) are presented in bold. *BMI* body mass index, *CVD* cardiovascular disease, *PGS* polygenic score.

# **Table S8.** Risk of CVD in relation to BMI category and a polygenic score for BMI, in the total sample and by sex.

| **Total sample** | **All** | **Men** | **Women** |
| --- | --- | --- | --- |
| **Independent effect model** |  |  |  |
| Overweight | **1·29 (1·20-1·39), p<0·001** | **1·30 (1·19-1·41), p<0·001** | **1·26 (1·13-1·42), p<0·001** |
| Obesity | **1·73 (1·53-1·96), p<0·001** | **1·69 (1·43-2·01), p<0·001** | **1·75 (1·47-2·08), p<0·001** |
| PGS_BMI_ | **1·11 (1·08-1·15), p<0·001** | **1·13 (1·08-1·18), p<0·001** | **1·09 (1·04-1·15), p<0·001** |
| **Joint effect model** |  |  |  |
| Overweight | **1·25 (1·16-1·35), p<0·001** | **1·25 (1·14-1·37), p<0·001** | **1·24 (1·11-1·40), p<0·001** |
| Obesity | **1·62 (1·43-1·85), p<0·001** | **1·57 (1·32-1·86), p<0·001** | **1·68 (1·40-2·00), p<0·001** |
| PGS_BMI_ | **1·07 (1·03-1·10), p<0·001** | **1·08 (1·03-1·13), p=0·002** | 1·04 (1·00-1·10), p=0·077 |
| **Interaction model** |  |  |  |
| Overweight | **1·24 (1·15-1·34), p<0·001** | **1·24 (1·13-1·36), p<0·001** | **1·23 (1·10-1·38), p<0·001** |
| Obesity | **1·81 (1·55-2·13), p<0·001** | **1·74 (1·44-2·09), p<0·001** | **1·88 (1·51-2·34), p<0·001** |
| PGS_BMI_ | **1·07 (1·02-1·12), p=0·007** | **1·08 (1·01-1·15), p=0·021** | 1·05 (0·98-1·12), p=0·147 |
| Overweight * PGS_BMI_ | 1·04 (0·96-1·12), p=0·305 | 1·03 (0·94-1·13), p=0·502 | 1·04 (0·94-1·17), p=0·440 |
| Obesity * PGS_BMI_ | **0·85 (0·75-0·98), p=0·022** | 0·86 (0·72-1·04), p=0·115 | 0·85 (0·71-1·01), p=0·063 |
| **By tertiles of the PGS BMI** |  |  |  |
| Overweight, low PGS | **1·19 (1·04-1·35), p=0·009** | **1·22 (1·04-1·43), p=0·015** | 1·14 (0·93-1·39), p=0·221 |
| Overweight, medium PGS | **1·22 (1·08-1·37), p=0·001** | **1·17 (1·00-1·37), p=0·047** | **1·29 (1·07-1·54), p=0·006** |
| Overweight, high PGS | **1·35 (1·19-1·53), p<0·001** | **1·39 (1·19-1·62), p<0·001** | **1·28 (1·06-1·53), p<0·001** |
| Obesity, low PGS | **2·15 (1·56-2·98), p<0·001** | **2·08 (1·37-3·15), p=0·001** | **2·16 (1·48-3·15), p<0·001** |
| Obesity, medium PGS | **1·82 (1·42-2·34), p<0·001** | **1·73 (1·30-2·30), p<0·001** | **1·91 (1·33-2·74), p<0·001** |
| Obesity, high PGS | **1·50 (1·27-1·77), p<0·001** | **1·49 (1·13-1·97), p=0·005** | **1·51 (1·19-1·91), p=0·001** |

Hazard rate ratios (95% confidence intervals) of CVD in relation to BMI category and the PGS_BMI_, for the total sample, separately for men and women. All models are adjusted for study, sex, smoking and education, and age used as the underlying time scale. Independent effect models contain either BMI category *or* PGS_BMI_ as predictors of CVD. Joint effect models contain BMI category *and* PGS_BMI_ together as predictors of CVD. Interaction models contain main effects of BMI category and the PGS_BMI_, and an interaction term between BMI category and the PGS_BMI_. Statistically significant estimates (at the α<0·05 level) are presented in bold. *BMI* body mass index, *CVD* cardiovascular disease, *PGS* polygenic score.

# References

1. Ludvigsson JF, Andersson E, Ekbom A, Feychting M, Kim JL, Reuterwall C, et al. External review and validation of the Swedish national inpatient register. BMC Public Health. 2011;11:450.

2. Brooke HL, Talback M, Hornblad J, Johansson LA, Ludvigsson JF, Druid H, et al. The Swedish cause of death register. Eur J Epidemiol. 2017;32(9):765-73.

3. Zagai U, Lichtenstein P, Pedersen NL, Magnusson PKE. The Swedish Twin Registry: Content and Management as a Research Infrastructure. Twin Res Hum Genet. 2019;22(6):672-80.

4. Finkel D, Pedersen N. Processing Speed and Longitudinal Trajectories of Change for Cognitive Abilities: The Swedish Adoption/Twin Study of Aging. Neuropsychology, development, and cognition Section B, Aging, neuropsychology and cognition. 2004;11(2):325-45.

5. Gold CH, Malmberg B, McClearn GE, Pedersen NL, Berg S. Gender and health: a study of older unlike-sex twins. J Gerontol B Psychol Sci Soc Sci. 2002;57(3):S168-76.

6. Gatz M, Fratiglioni L, Johansson B, Berg S, Mortimer JA, Reynolds CA, et al. Complete ascertainment of dementia in the Swedish Twin Registry: the HARMONY study. Neurobiology of Aging. 2005;26(4):439-47.

7. McCarthy S, Das S, Kretzschmar W, Delaneau O, Wood AR, Teumer A, et al. A reference panel of 64,976 haplotypes for genotype imputation. Nat Genet. 2016;48(10):1279-83.

8. Lloyd-Jones LR, Zeng J, Sidorenko J, Yengo L, Moser G, Kemper KE, et al. Improved polygenic prediction by Bayesian multiple regression on summary statistics. Nat Commun. 2019;10(1):5086.

9. Yengo L, Sidorenko J, Kemper KE, Zheng Z, Wood AR, Weedon MN, et al. Meta-analysis of genome-wide association studies for height and body mass index in ∼700000 individuals of European ancestry. Human Molecular Genetics. 2018;27(20):3641-9.

10. Wray NR, Lee SH, Mehta D, Vinkhuyzen AAE, Dudbridge F, Middeldorp CM. Research Review: Polygenic methods and their application to psychiatric traits. Journal of Child Psychology and Psychiatry. 2014;55(10):1068-87.
